# Developmental mapping of small-conductance calcium-activated potassium channel expression in the rat nervous system

**DOI:** 10.1002/cne.23466

**Published:** 2014-02-12

**Authors:** Marco Gymnopoulos, Lorenzo A Cingolani, Paola Pedarzani, Martin Stocker

**Affiliations:** 1 Department of Molecular Biology of Neuronal Signals, Max Planck Institute for Experimental Medicine37075, Göttingen, Germany; 2 Department of Neuroscience and Brain Technologies, Istituto Italiano di Tecnologia (IIT)16163, Genoa, Italy; 3 Research Department of Neuroscience, Physiology and Pharmacology, University College LondonLondon, WC1E 6BT, United Kingdom

**Keywords:** SK channel, in situ hybridization, embryonic and postnatal distribution, SK1, SK2, and SK3 ontogenesis, afterhyperpolarizing current, apamin

## Abstract

Early electrical activity and calcium influx regulate crucial aspects of neuronal development. Small-conductance calcium-activated potassium (SK) channels regulate action potential firing and shape calcium influx through feedback regulation in mature neurons. These functions, observed in the adult nervous system, make them ideal candidates to regulate activity-and calcium-dependent processes in neurodevelopment. However, to date little is known about the onset of expression and regions expressing SK channel subunits in the embryonic and postnatal development of the central nervous system (CNS). To allow studies on the contribution of SK channels to different phases of development of single neurons and networks, we have performed a detailed in situ hybridization mapping study, providing comprehensive distribution profiles of all three SK subunits (SK1, SK2, and SK3) in the rat CNS during embryonic and postnatal development. SK channel transcripts are expressed at early stages of prenatal CNS development. The three SK channel subunits display different developmental expression gradients in distinct CNS regions, with time points of expression and up-or downregulation that can be associated with a range of diverse developmental events. Their early expression in embryonic development suggests an involvement of SK channels in the regulation of developmental processes. Additionally, this study shows how the postnatal ontogenetic patterns lead to the adult expression map for each SK channel subunit and how their coexpression in the same regions or neurons varies throughout development. J. Comp. Neurol. 522:1072–1101, 2014. © 2013 Wiley Periodicals, Inc.

The development of neurons and neural networks involves a sequential interplay between gene expression controlled by transcription factors and patterned activity mediated by the orchestrated action of receptors and ion channels (for review see Ben-Ari and Spitzer, 2010). In this context, a prominent role is played by transient calcium (Ca^2+^) elevations driven by different Ca^2+^-permeable ligand-and voltage-gated channels. Ca^2+^ influx, characterized by different spatiotemporal patterns depending on the developmental stage, regulates various aspects of neuronal development, including the proliferation of neural progenitors (LoTurco et al., 1995; Nacher and McEwen, 2006; Dave and Bordey, 2009), neuronal migration (Komuro and Rakic, 1996; Bortone and Polleux, 2009), axon guidance (Hong et al., 2000; Li et al., 2005; Shim et al., 2005; Wang and Poo, 2005), neurotransmitter and receptor specification (Borodinsky et al., 2004; for review see Spitzer et al., 2004; Spitzer, 2006), and synapse formation (Chudotvorova et al., 2005; Akerman and Cline, 2006; Wang and Kriegstein, 2008).

After its influx into neurons, Ca^2+^ activates various Ca^2+^-dependent ion channels with modalities and dynamics that depend on their association with the Ca^2+^ sources in tighter or looser domains (for review see Fakler and Adelman, 2008). In particular, Ca^2+^-dependent K^+^ channels couple intracellular Ca^2+^ elevations to K^+^ efflux and hyperpolarization of the membrane potential, thereby dampening membrane excitability. Among them, small-conductance Ca^2+^-activated K^+^ channels of the SK (K_Ca_2) family are functionally coupled to Ca^2+^ sources (e.g., voltage-gated Ca^2+^ channels, IP_3_ receptors) in various neuronal compartments, where they shape the spatiotemporal dynamics of intracellular Ca^2+^ transients through feedback mechanisms (Cai et al., 2004; Faber et al., 2005; Ngo-Anh et al., 2005; Bloodgood and Sabatini, 2007; Faber, 2010; Giessel and Sabatini, 2010; Tonini et al., 2013). Given the importance of the spatiotemporal patterns of Ca^2+^ elevations and patterned electrical activity in neuronal ontogenesis, SK channels are therefore prime candidates as potential regulators of various Ca^2+^-dependent processes in neurodevelopment.

Molecular cloning has identified three members of the SK channel family, SK1 (K_Ca_2.1), SK2 (K_Ca_2.2), and SK3 (K_Ca_2.3; Kohler et al., 1996; Joiner et al., 1997; Chandy et al., 1998). They are all voltage-insensitive, gated by Ca^2+^ binding to calmodulin, which is constitutively bound to their carboxy-terminal region (for review see Fakler and Adelman, 2008). By responding to changes in intracellular Ca^2+^, SK channels directly link Ca^2+^ signals to membrane excitability and modulation of electrical activity in neurons. SK channels are expressed in several tissues and prominently throughout the adult central nervous system (CNS; Stocker and Pedarzani, 2000; Tacconi et al., 2001; Sailer et al., 2004). In the mature CNS, the three SK channel subunits have partially overlapping but clearly distinct distribution patterns, with SK1 and SK2 displaying extensive colocalization and SK3 presenting a complementary distribution (Stocker and Pedarzani, 2000; Tacconi et al., 2001; Sailer et al., 2004). Several studies suggest that specific SK subunits contribute to adult neuronal excitability and function in different brain regions and possibly, on a cellular level, in different neuronal compartments (for review see Pedarzani and Stocker, 2008; Adelman et al., 2012).

However, to date, the onset of expression and detailed mapping of SK channel distribution in the CNS during embryonic and postnatal development are missing. This information is essential to establish the contribution of SK channels to different phases of development of single neurons and networks. The aim of the present study is to provide comprehensive distribution profiles of all three SK subunits in the rat CNS during embryonic and postnatal development, to determine their onset of expression and the ontogenetic patterns leading to the adult expression maps.

## MATERIALS AND METHODS

### Northern blot

Poly(A)^+^ RNA was isolated from heads (E12 and E13) or whole-brain tissue (E15, E17, E19, E21, P1, P3, P6, P12, and P24) using oligo(dT) cellulose (FastTrack 2.0; Invitrogen, Carlsbad, CA). RNA concentrations were estimated with a fluorescence-based RNA quantitation assay (RiboGreen; Invitrogen), and 5 μg RNA was separated for each developmental time point by formaldehyde agarose (1%) gel electrophoresis and transferred to a positively charged membrane (Roche Applied Sciences, Indianapolis, IN). Membranes were stained with methylene blue to control for even transfer of RNA.

**Figure 1:**
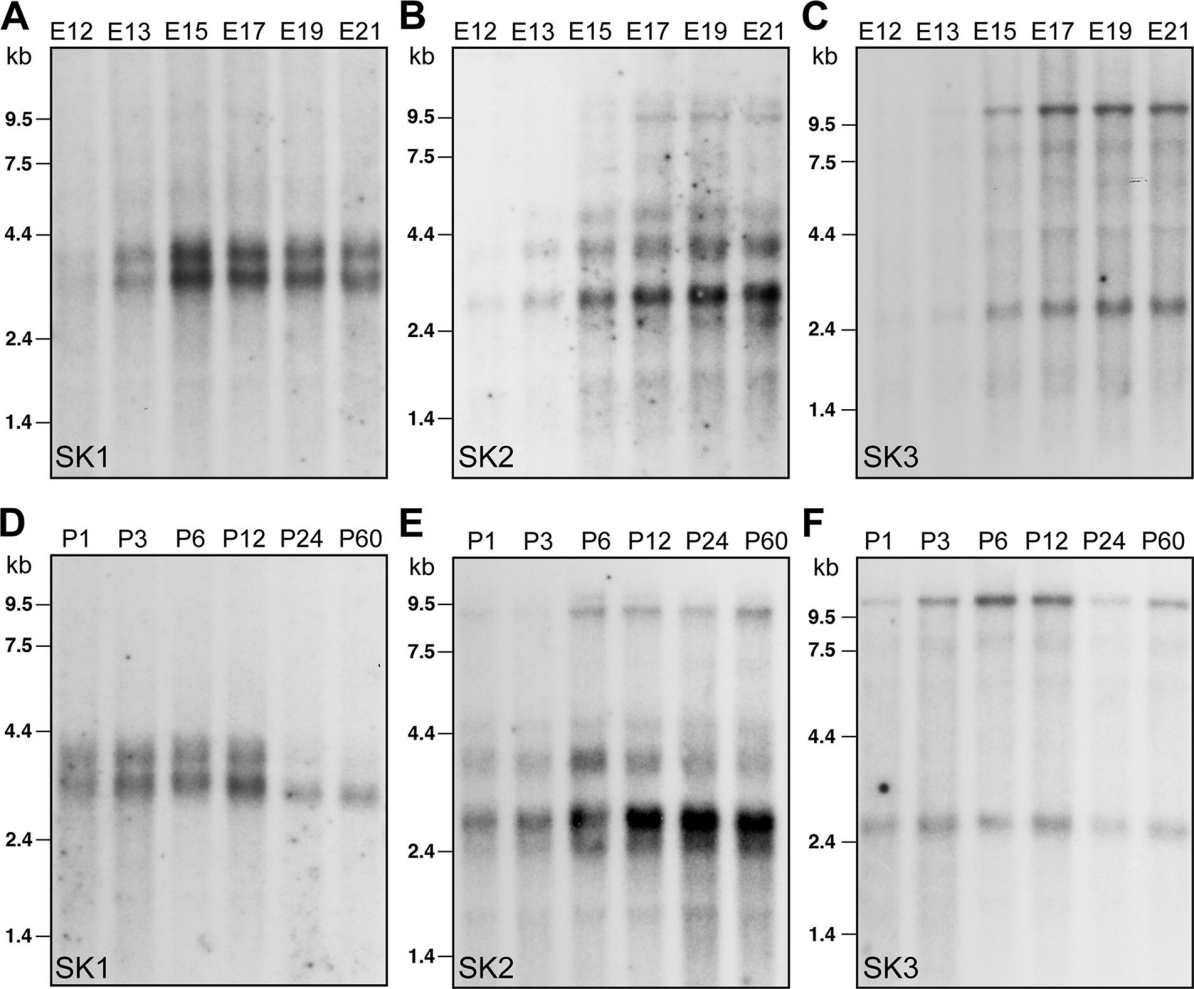
Northern blots displaying SK channel subunit transcripts at different stages of embryonic (A–C) and postnatal (D–F) development. Two bands 3.2 kb and 4.0 kb in length were observed for SK1 (A,D), two main bands of 2.8 kb and 3.6 kb for SK2 (B,E) and two bands of 2.6 kb and 10 kb for SK3 (C,F).

To generate probes specific for each SK channel subunit, sequences spanning the carboxy-terminus and the 3′-untranslated region for SK1 and SK2 were identified in cDNA clones and confirmed by rapid amplification of cDNA ends (3′ RACE; Invitrogen). For SK3, a specific probe was generated from a region within the extended amino-terminus. The nucleotide ranges used to generate the three probes are given after the GenBank accession numbers: SK1: NW_003812064, 941,552–942,545 nt, SK2: NW_003812674, 312,752–313,332 nt, SK3: NM_019315, 553–1,075. The Northern was probed with ^32^P-dCTP labeled fragments (2 × 10^6^ cpm/ml) in 10 ml ExpressHyb hybridization solution (Clontech Laboratories, Palo Alto, CA) for 1 hour. The final wash was for 30 minutes in 0.1–0.2× SET (1xSET is: 150 mM NaCl, 20 mM Tris-HCl, 1 mM EDTA, pH 7.5) and 0.3% sodium dodecyl sulfate (SDS) at 60–68°C. Autoradiography was performed with an intensifying screen at −80°C. Exposure times were adjusted for each blot to obtain the optimal signal-to-noise ratio, prevent saturation of signals at highly expressing developmental stages, and stay within the linear range of the intensifying screen (see Fig. 1A–F). To appreciate the changes occurring across the developmental stages, it is important to comprehend that the signal intensity of bands at E21 and P1 reflects comparable RNA levels.

### Tissue preparation

All procedures involving the use of animals were approved by the local animal care committee according to current laws for animal protection. Data were obtained from at least three unrelated animals for each embryonic and postnatal time point. Timed matings for Wistar rats were set up in house, and embryonic (E11, E12, E13, E15, E17, E19, E21) and postnatal (P1, P3, P6, P12, P24) stages were used. Rats were paired for 3 hours early in the morning, and E1 was defined as 24 hours after impregnation. P1 was defined as 24 hours after birth. The mother or pups were terminally anesthetized and whole embryos (E1–E17), heads (E19 and E21), or brains (P1–P24) were removed and frozen on powdered dry ice (Wisden and Morris, 1994). To allow a detailed comparative analysis, sagittal and horizontal sections (10–16 μm) were cut with a cryostat, thaw mounted onto silan-coated slides (3′-aminopropyltriethoxysilan; Sigma, St. Louis, MO) and air dried. After 10–30 minutes of fixation in 4% paraformaldehyde dissolved in phosphate-buffered saline (PBS; 130 mM NaCl, 7 mM disodium hydrogen phosphate, 3 mM sodium dihydrogen phosphate, pH 7.4), slides were washed in PBS and dehydrated in an ascending ethanol series. Sections were stored in 100% ethanol at 4°C until use.

### Oligonucleotide probes

Selection of two independent antisense oligonucleotides for each the three channels subunits SK1, SK2, and SK3 has been described by Stocker and Pedarzani (2000). The sequences of the 45-nucleotide-long oligonucleotides were as follows: SK1 (K_Ca_2.1, KCNN1): 5′-GGC CTG CAG CTC CGA CAC CAC CTC ATA TGC GAT GCT CTG TGC CTT-3′, 5′-CAG TGG CTT TGT GGG CTC TGG GCG GCT GTG GTC AGG TGA CTG GGC-3′; SK2 (K_Ca_2.2, KCNN2): 5′-AGC GCC AGG TTG TTA GAA TTG TTG TGC TCC GGC TTA GAC ACC ACG-3′, 5′-CTT CTT TTT GCT GGA CTT AGT GCC GCT GCT GCT GCC ATG CCC GCT-3′; and SK3 (K_Ca_2.3, KCNN3): 5′-CGA TGA GCA GGG GCA GGG AAT TGA AGC TGG CTG TGA GGT GCT CCA-3′, 5′-TAG CGT TGG GGT GAT GGA GCA GAG TCT GGT GGG CAT GGT TAT CCT-3′.

Oligonucleotides were 3′ end labeled with [α^35^S]dATP (1,250 Ci/mmol; PerkinElmer/NEN, Boston, MA) or [α^35^S]dATP (1,200 Ci/mmol; Amersham Biosciences, Arlington Heights, IL) using terminal deoxynucleotidyl transferase (Roche, Mannheim, Germany); unincorporated nucleotides were removed using Bio-Spin 6 columns (Bio-Rad, Hercules, CA). For hybridization, 4 × 10^5^ cpm (∼2–5 pg/ml) of each probe per slide was used.

### In situ hybridization

Hybridization was performed as described by Stocker and Pedarzani (2000). In brief, slides were air dried and hybridized overnight at 42°C in 100 μl solution containing 50% formamide, 10% dextran sulfate, 50 mM dithiothreitol (DTT), 300 mM NaCl, 30 mM Tris-HCl (pH 7.4), 4 mM EDTA, 1× Denhardt's solution (Sigma), 0.5 mg/ml acid/alkali denatured salmon sperm DNA, and 0.5 mg/ml polyadenylic acid (Sigma). After hybridization, slides were washed twice for 20 minutes each in 1× SSC containing 50 mM β-mercaptoethanol, for 45 minutes in 1× SSC at 57°C, and for 5 minutes in 1× SSC, followed by 5 minutes in 0.1× SSC at room temperature. Sections were once again dehydrated in an ascending ethanol series, air dried, and exposed to Kodak Biomax MR X-ray film (Eastman Kodak, Rochester, NY) for 14 days. For cellular resolution, slides were subsequently dipped in photographic emulsion Kodak NTB, incubated for 12–20 weeks at 4°C, and then developed in Kodak D-19 for 3.5 minutes. Sections were counterstained with 0.1% cresyl violet (Nissl stain) to confirm cytoarchitecture and analyzed via bright-and darkfield microcopy. Rat brain regions were identified according to Paxinos et al. (1994), Altman and Bayer (1995), and Paxinos and Watson (1998).

### Data analysis

**Figure 2:**
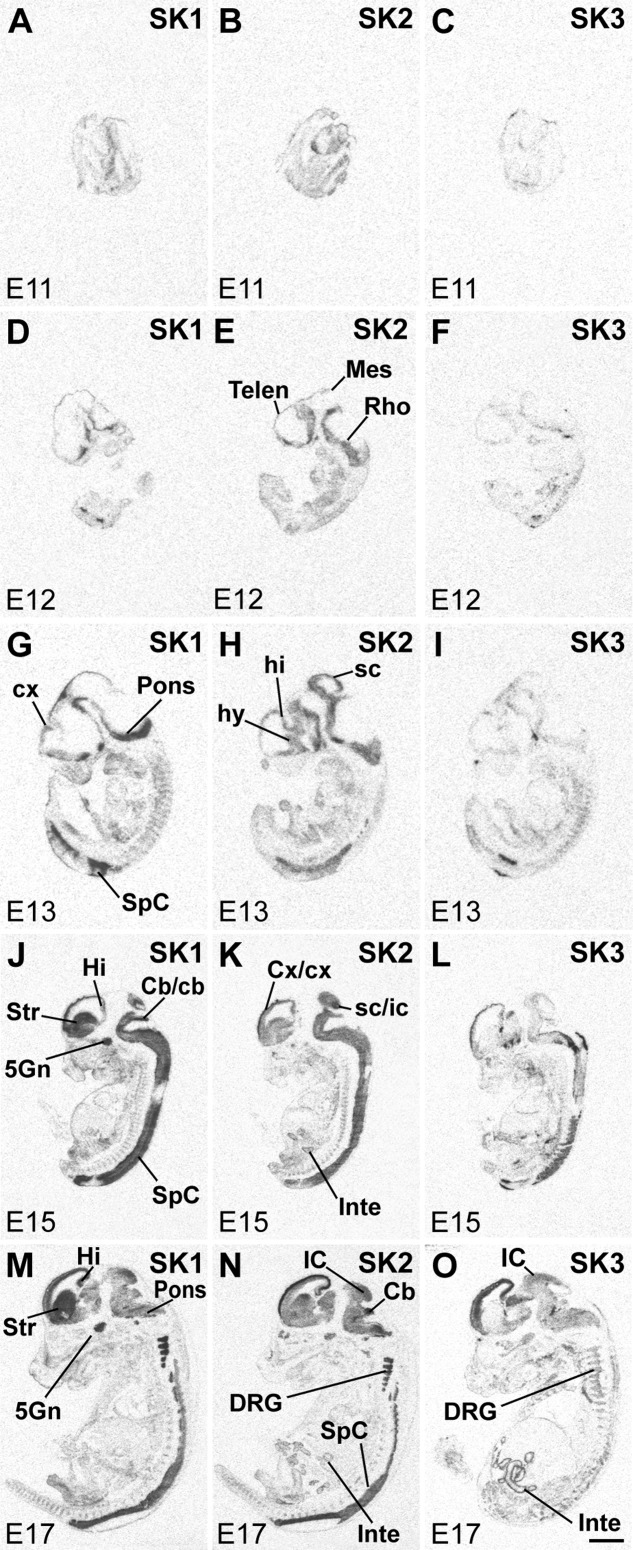
SK subunit mRNA expression in rat embryos (E11: A–C; E12: D–F; E13: G–I; E15: J–L; and E17: M–O). X-ray film images of sections hybridized with oligonucleotide probes specific for SK1 (left column), SK2 (middle column) and SK3 (right column) show distribution of transcripts in the CNS and the body. Dark areas contain high levels of mRNA. For abbreviations see list. Abbreviations are according to Paxinos et al. (1994) and Paxinos and Watson (1998). Scale bar = 2 mm.

**Figure 3:**
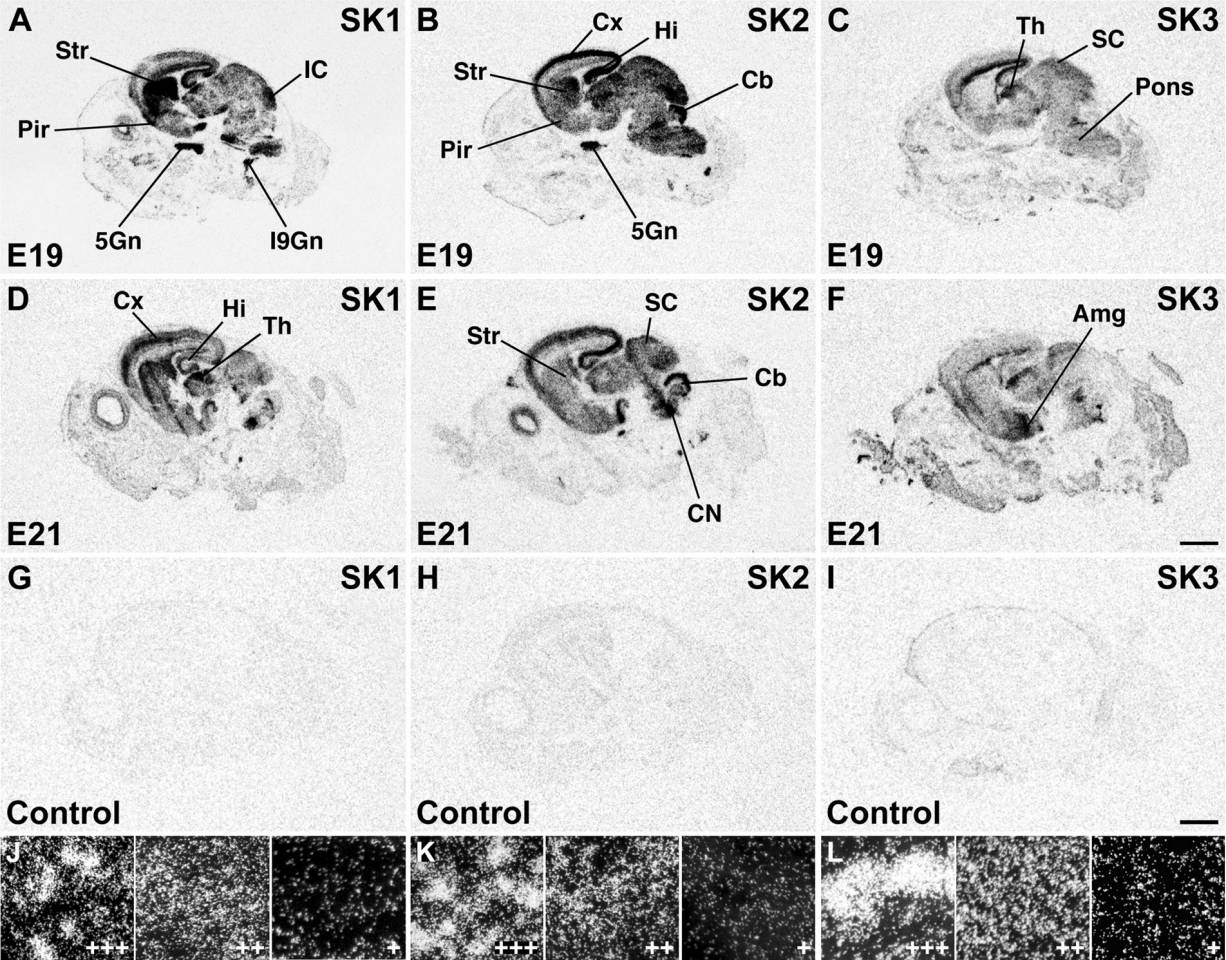
X-ray images of sagittal sections of rat heads at E19 (A–C) and E21 (D–F) show largely overlapping distribution of SK1 and SK2 transcripts in several cortical and subcortical areas, whereas SK3 mRNA has a complementary distribution pattern. Control sections at E21 (G–I) hybridized in the presence of 100-fold excess cold oligonucleotide for each probe show a uniformly low, nonspecific labeling. Darkfield photomicrographs from various brain regions at E19–E21 exemplifying signals of strong (+++), moderate (++), and weak (+) intensity for SK1 (J), SK2 (K), and SK3 (L). For abbreviations see list. Scale bars = 2 mm in F (applies to A–F); 2 mm in I (applies to G–I).

Autoradiograms (see Figs. 2, 3, 7) provided an overview of the distribution of the three SK channel subunits in different areas of the developing rat brain. Analysis was performed on emulsion-dipped slides to resolve cellular labeling. All slides were emulsion coated, and the weak and strong signals in various brain regions shown in Tables6 were quantified according to the relative silver grain density on the individual cell bodies obtained with a given oligonucleotide. Expression levels were assessed by two independent observers. The scoring is the result of their joint assessment. The results of this analysis are presented in Tables6 as −, +, ++, or +++, with − representing signals below the threshold limit of detection and + a weak, ++ a moderate, and +++ the strongest level of expression. A scale of signal intensities with representative examples for each SK channel subunit is shown in Figure 3J–L. “Not clearly identifiable” (•) signifies that a given region could not be observed in our sections or could not be clearly differentiated from adjacent regions.

**Table 1:** Prenatal Distribution (E15–E21) of the Small-Conductance Calcium-Activated Potassium Channel Subunit SK1^1^

Brain region	E15	E17	E19	E21
**Olfactory system**				
Olfactory bulb				
Glomerular layer	np	np	−	−
Mitral cell layer	np	np	−	−
Internal granular layer	np	np	np	+
Accessory olfactory bulb	np	−	−	−
Anterior olfactory nucleus	np	•	++	+++
Olfactory tubercle	np	np	++	+++
**Cerebral cortex**				
Piriform cortex	•	•	++	++
Primordial plexiform layer	+++	np	np	np
Cortical neuroepithelium	−	−	−	−
Molecular layer	np	+	+	+
Cortical plate	np	++	++	++
Intermediate zone	np	+	+	+++
Subventricular zone	np	++	++	−
Hippocampal formation				
Subicular neuroepithelium	+	++	np	np
Subiculum (or diff. field)	np	++	++	+++
Hippocampal neuroepithelium	+	++	np	np
Hippocampus (or diff. field)	+++	+++	np	np
CA1	np	np	+++	+++
CA3	np	np	+++	+++
Dentate gyrus granule cells	np	np	−	+
**Basal ganglia**				
Striatum (or diff. field)	+++	+++	+++	+++
**Thalamus**				
Anterior thalamus (or diff. field)	−	++	+++	+++
Posterior thalamus (or diff. field)	−	+	+	+
Intermediate thalamus (or diff. field)	−	•	++	++
**Trigeminal ganglion**	+++	+++	+++	+++
**Inferior glossopharyngeal ganglion**	•	•	+++	+++
**Pons (or diff. field)**	++	++	+++	•
**Cerebellum**				
Cerebellar neuroepithelium	−	−	np	np
Cerebellum (or diff. field)	++	++	np	np
Deep nuclei	np	np	+++	+++
Purkinje cell layer	np	np	−	−
External germinal layer	np	−	−	−
Granule cell layer	np	np	−	−
**Spinal cord**	+++	+++	•	•
**Dorsal root ganglia**	+++	+++	•	•
**Intestine**	−	−	•	•

1 +++, strong; ++, moderate; +, weak; −, signals very low or below the threshold limit of detection; np, not present; •, not clearly identifiable; Diff. field, differentiating field.

The specificity of the oligonucleotides used to study the distribution of SK1, SK2, and SK3 was checked in several control experiments. Hybridized sections showed identical patterns, with each pair of oligonucleotides used for each of the three genes. To determine nonspecific hybridization to sections, adjacent sections were hybridized with sense oligonucleotides. As a further control for specificity, the background signal was assessed by competition hybridizations (examples of which are shown in Figs. 3G–I, 5K,L, 6K,L 7K,L), in which radioactively labeled probes were hybridized to sections in the presence of excess (100-fold) unlabeled (cold) probe. All these experiments displayed minimal levels of nonspecific binding, barely distinguishable from the overall background (dipping of nonhybridized sections).

Several factors other than transcript levels (i.e., hybridization efficiency of individual probes, intrinsic properties of tissue from different brains) can affect signal intensity. Therefore, this scoring reflects relative amounts of each transcript in different brain areas rather than comparisons among the three different SK transcripts. However, the fact that we could observe similar absolute signal intensities (in particular in the strongest labeling range) with probes for each SK subunit gene in different brain regions suggests that the influence of factors such as the hybridization efficiency was minor. Therefore, relative levels of the three different SK mRNAs may cautiously be compared.

## RESULTS

### Analysis of SK-subunit mRNA levels in the developing rat CNS

The developmental regulation of the expression of SK channel subunits in the rat brain was first studied by Northern blot analysis (Fig. 1). Northern blots for the SK1, SK2, and SK3 channel transcripts isolated from rat embryos at prenatal stages E12–E21 showed how early SK subunit mRNAs were detectable in the CNS. SK1 and SK2 transcripts were expressed weakly, if at all, at E12 but were clearly detectable at E13 (Fig. 1A,B). By contrast, the SK3 mRNA levels could first be detected at E15 and were weak if at all detectable at E12 and E13.

Throughout development, two bands 3.2 kb and 4.0 kb in length were observed for SK1 (Fig. 1A,D). Expression levels for SK1 mRNA decreased from a peak value at E15 until P60 (Fig. 1A,D). The most drastic change was observed at P24, when mainly the 3.2-kb band of a substantially lower intensity was left, which persisted in adulthood (Fig. 1D). There were also two main transcripts (2.8 and 3.6 kb) for SK2, which showed different time courses of expression during development (Fig. 1B,E). The 2.8-kb transcript increased gradually throughout embryonic and postnatal development, doubling between P3 and P12 and reaching its maximal expression at P24 (Fig. 1B,E). By contrast, the 3.6-kb transcript increased up to P6, when the strongest signal intensity was observed, and declined thereafter (Fig. 1B,E). The two transcripts (2.6 and 10 kb) observed for SK3 increased throughout development, reaching maximal levels at P12 (Fig. 1C,F). As in the case of SK1, the SK3 expression levels decreased thereafter (Fig. 11F). These results show that SK channel mRNAs are expressed in the brain already at very early stages of prenatal development and that their levels are subject to developmental regulation with a pattern that is specific for each SK channel subunit.

### Embryonic distribution of SK1, SK2, and SK3 transcripts: overview

**Figure 4:**
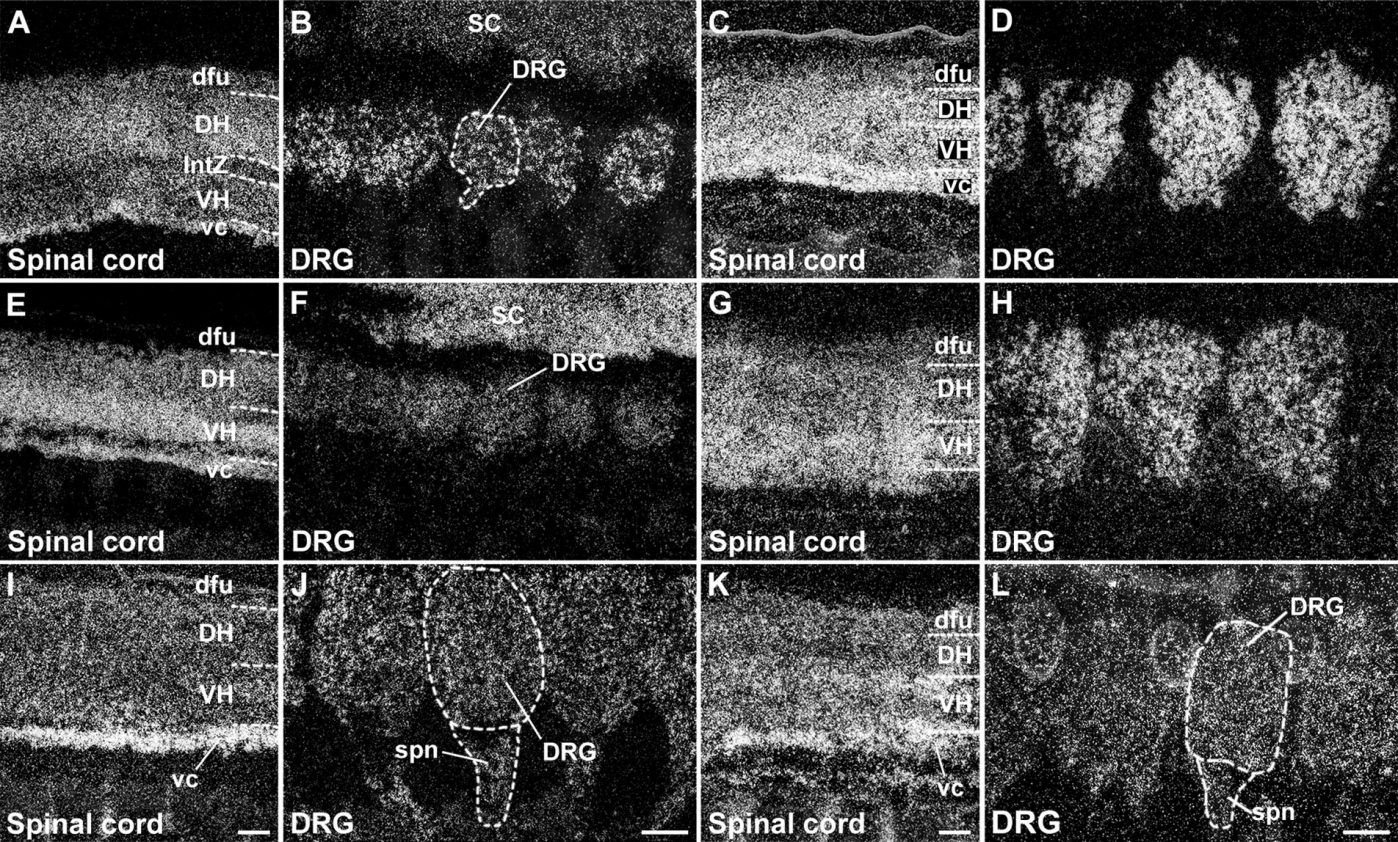
Distribution of SK channel transcripts in the spinal cord at E15 (A,E,I) and E17 (C,G,K) and in the dorsal root ganglia (DRG) at E15 (B,F,J) and E17 (D,H,L). The darkfield photomicrographs show that all three SK channel transcripts are expressed in both dorsal (DH) and ventral horn (VH) neurons of the embryonic spinal cord (SK1: A,C; SK2: E,G; SK3: I,K) and in dorsal root ganglia neurons (SK1: B,D; SK2: F,H; SK3: J,L). Bright areas contain high levels of mRNA. SK3 transcripts are also present in spinal nerves (spn, J,L). For abbreviations see list. Scale bars = 150 μm in I (applies to A,E,I); 150 μm in J (applies to B,F,J); 150 μm in K (applies to C,G,K); 150 μm in L (applies to D,H,L).

The results of the Northern analysis raised the question of whether the developmental changes in the SK expression levels reflect changes in their distribution patterns before birth. To address this question, we performed a detailed in situ hybridization analysis and mapped the distributions of SK1, SK2, and SK3 mRNAs in the rat embryonic nervous system. An overview of the prenatal distribution of the SK channel subunits was obtained by examination of X-ray film images (Figs. 2, 3), and, for cellular resolution, emulsion-coated sections were analyzed (Figs. 4, 8, 9, 11, 3, Tables3). Overview pictures of whole embryos (E11–E17; Fig. 2) and embryonic heads (E19–E21; Fig. 3) were obtained after hybridization with radioactively labeled oligonucleotides specific for each of the three SK channel subunits. Criteria for strong vs. weak labeling of brain structures were the number of silver grains accumulated above cell somata relative to the strongest hybridization signal for each given oligonucleotide (see also Fig. 3J–L).

**Figure 5:**
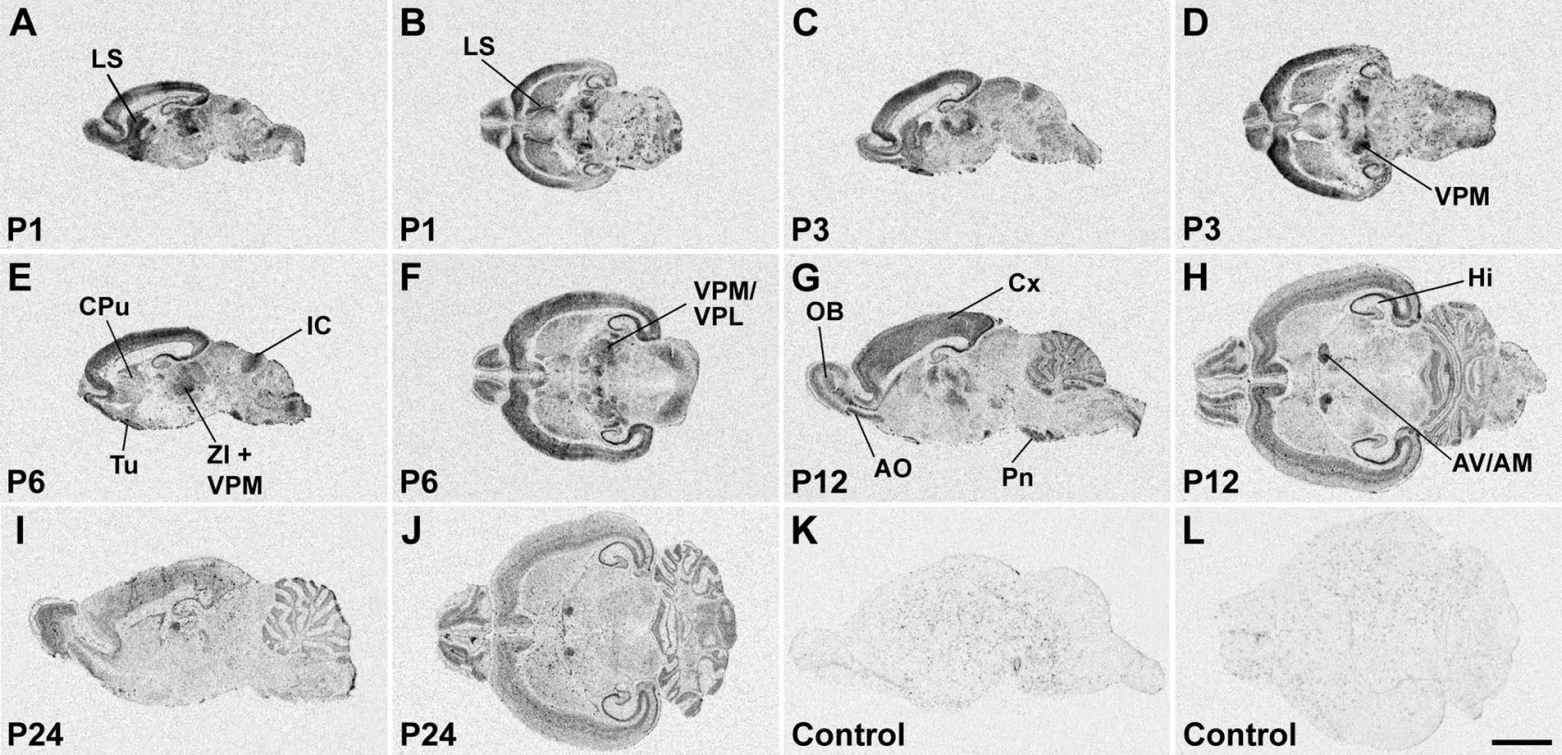
Distribution of SK1 channel subunit mRNA in the CNS at postnatal stages P1, P3, P6, P12, and P24 is shown in X-ray images of sagittal (A,C,E,G,I,K) and horizontal (B,D,F,H,J,L) sections hybridized with an oligonucleotide probe specific for SK1. Strong hybridization signals are present in the anterior olfactory nucleus (AO) and olfactory tubercle (Tu), cerebral cortex (Cx), hippocampal formation (Hi), and several thalamic nuclei. For details on the distribution see Table4. Control sections (K,L) were hybridized in the presence of 100-fold excess cold oligonucleotide, showing a uniformly low background signal. For abbreviations see list. Scale bar = 4 mm.

**Figure 6:**
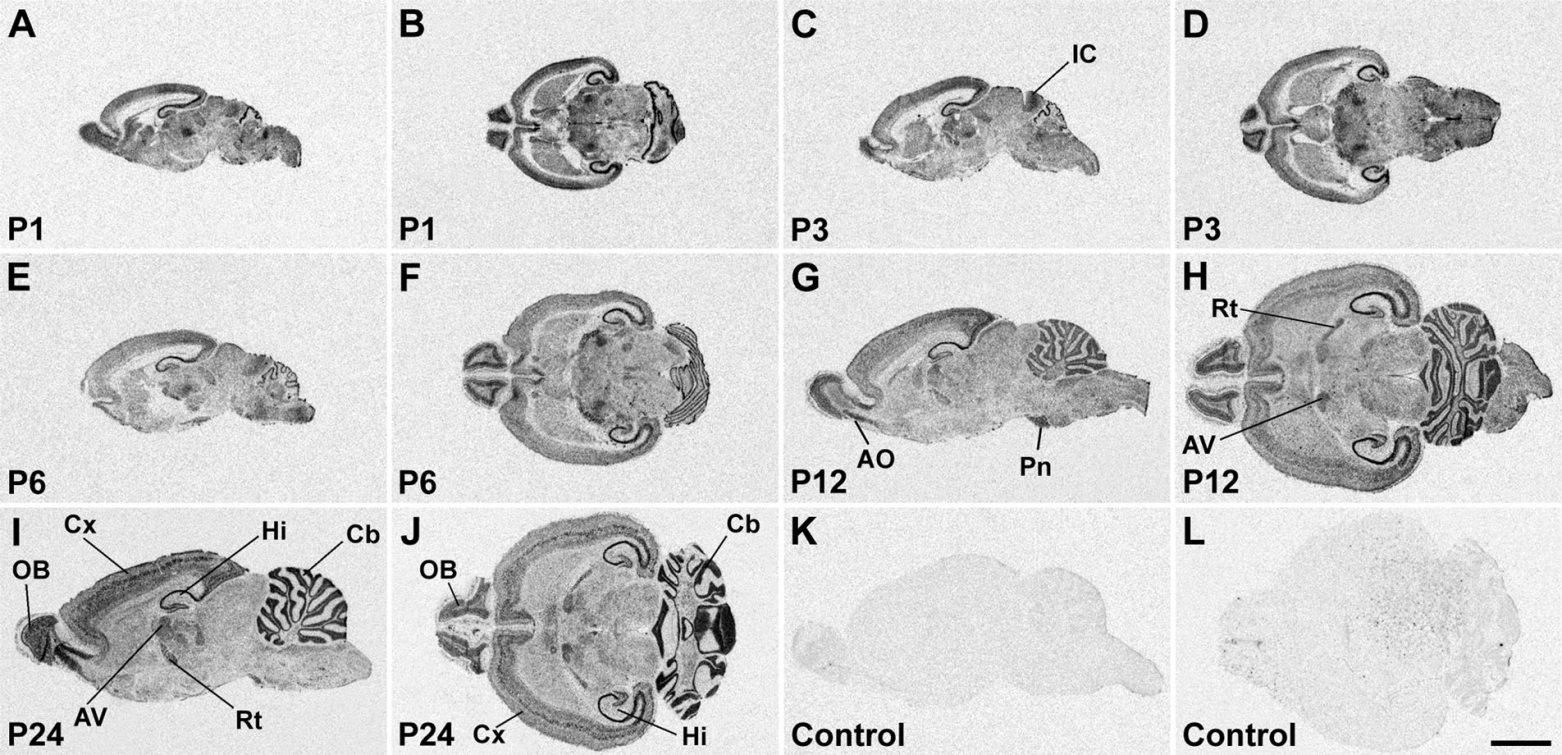
SK2 subunit mRNA expression in the postnatal rat brain. X-ray images of sagittal (A,C,E–G,I,K) and horizontal (B,D,H,J,L) sections at postnatal stages P1, P3, P6, P12, and P24 showing strong SK2 hybridization signals in the olfactory system, cerebral cortex (Cx), hippocampus (Hi), and cerebellum (Cb). For details on the distribution see Table5. Control sections (K,L) were hybridized in the presence of 100-fold excess cold oligonucleotide, showing a uniformly low, nonspecific labeling. For abbreviations see list. Scale bar = 4 mm.

**Figure 7:**
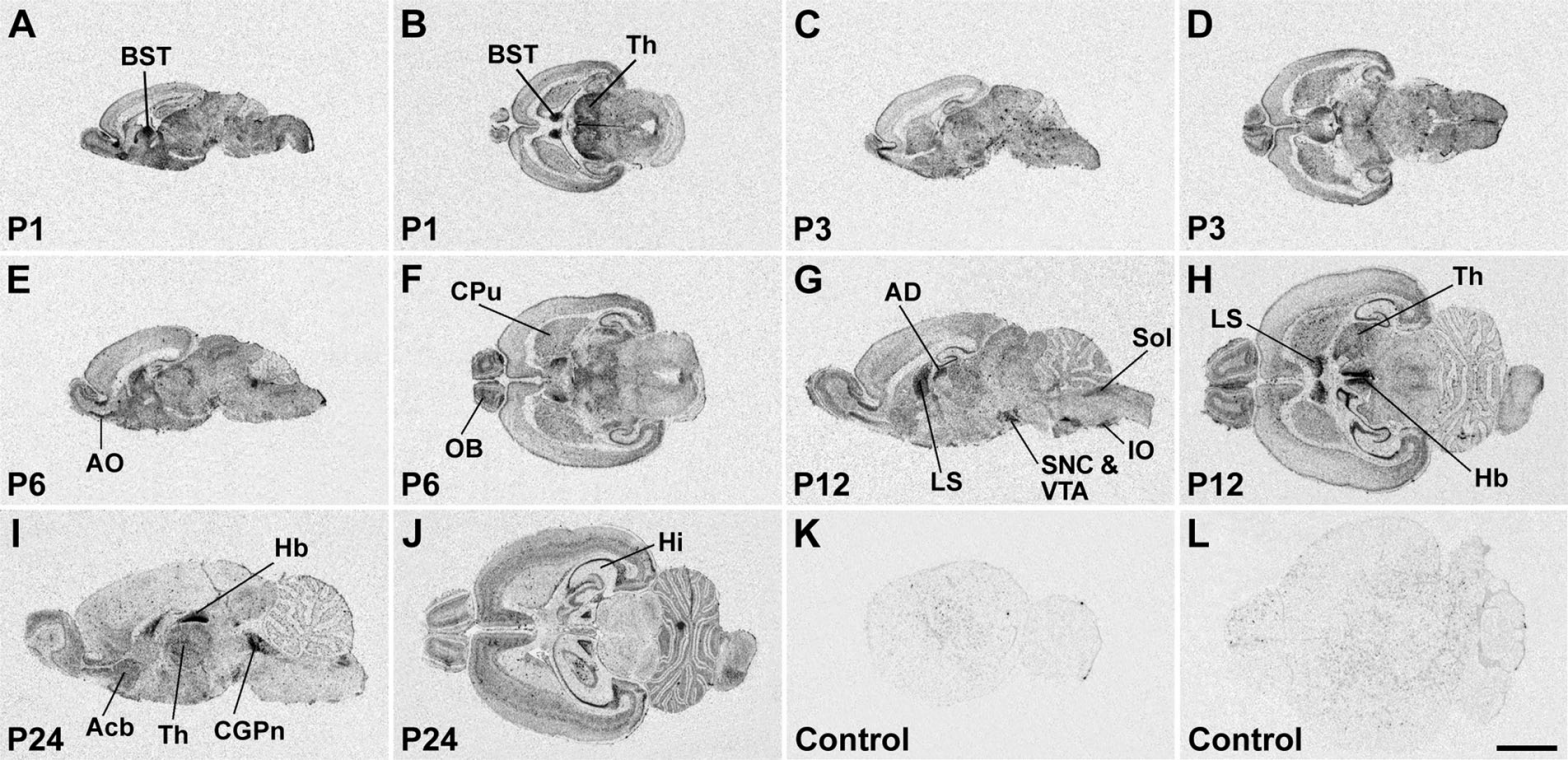
X-ray images of sagittal (A,C,E,G,I,K) and horizontal (B,D,F,H,J,L) sections hybridized with an oligonucleotide probe specific for SK3 show the distribution of this channel subunit in the rat CNS at postnatal stages P1, P3, P6, P12, and P24. The hybridization pattern differs substantially from the patterns of SK1 (Fig. 5) and SK2 (Fig. 6) and shows strong SK3 mRNA expression in the accessory olfactory bulb, anterior olfactory nucleus (AO) and olfactory tubercle, hippocampus (Hi), basal ganglia, septum, amygdala, thalamus (Th), and several hypothalamic and brainstem nuclei. For details on the distribution see Table6. Control sections (K,L) hybridized in the presence of 100-fold excess cold oligonucleotide show a low and uniform background signal. For abbreviations see list. Scale bar = 4 mm.

**Figure 8:**
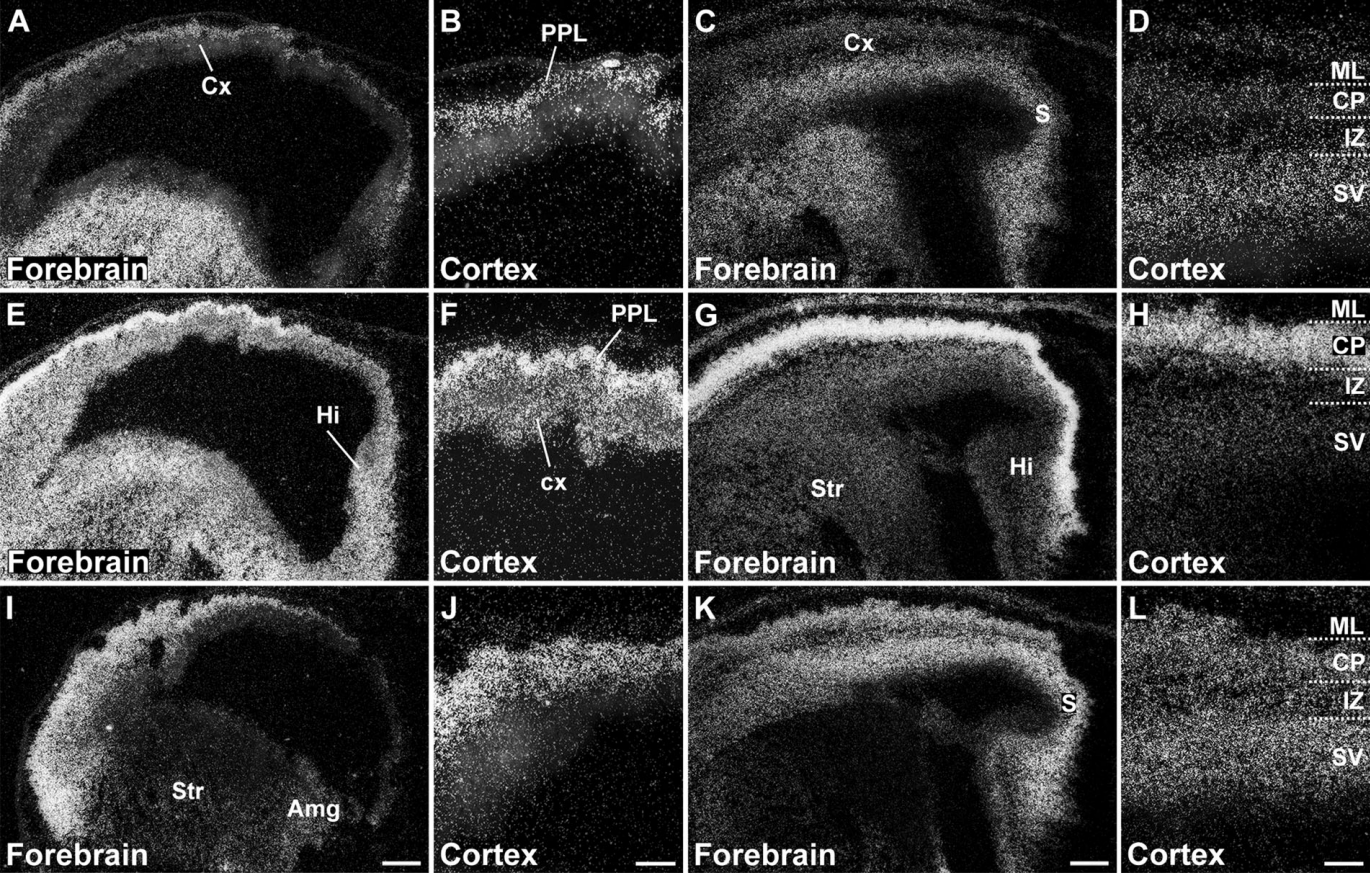
Differential expression of SK1 (A–D), SK2 (E–H), and SK3 (I–L) in the embryonic forebrain and cerebral cortex at stages E15 (A,B,E,F,I,J) and E17 (C,D,G,H,K,L). The darkfield photomicrographs show that, at E15, SK2 is strongly expressed in the primordial plexiform layer (PPL; E,F), together with a weaker expression of SK1 (A,B), and in the cortical neuroepithelium (cx). SK3 shows a more diffuse expression throughout the differentiating cortex (I,J). Strong expression of SK1 is visible in the striatal differentiating field (Str), with a weaker one in the adjacent striatal neuroepithelium lining the lateral ventricle (A). SK2 is strongly expressed in both striatal neuroepithelium and differentiating field (E). By contrast, SK3 is hardly detectable in the striatum but is expressed at weak to moderate levels in the adjacent differentiating amygdala (Amg; I). At E17, all three channels are present in the subiculum (S) and hippocampal primordium (Hi; C,G,K). Cortical expression of the three SK channel transcripts at E17 is shown in D,H,L, with SK2 displaying maximal expression levels in the cortical plate (CP; H). For details on the distribution see Tables3. For abbreviations see list. Scale bars = 250 μm in I (applies to A,E,I); 100 μm in J (applies to B,F,J); 250 μm in K (applies to C,G,K); 100 μm in L (applies to D,H,L).

**Figure 9:**
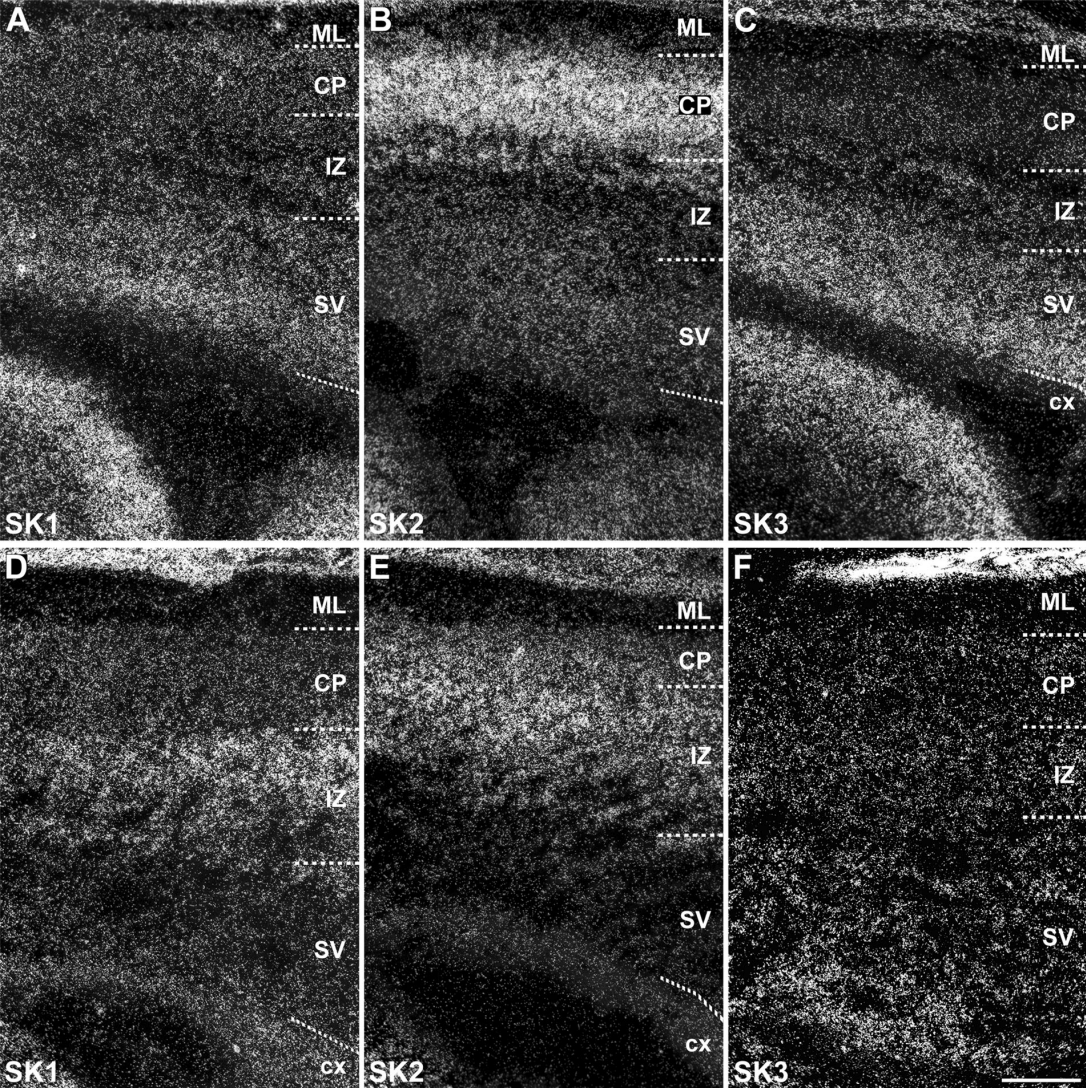
Distribution of SK1, SK2, and SK3 subunit transcripts in the immature embryonic neocortex showing changes in the distribution of the three subunits in the transition from stage E19 (A–C) to E21 (D–F). In particular, SK1 (A,D) decreases in the subventricular zone (SV) and increases in the intermediate zone (IZ); SK2 (B,E) displays a similar decrease of expression in the subventricular zone but also in the cortical plate (CP), accompanied by an increase in the intermediate zone; finally, SK3 shows a decreased expression in the subventricular zone at E21 (C,F). For abbreviations see list. For details on the distribution see Tables3. Scale bar = 200 μm.

The three SK channel subunits present clearly distinct distribution patterns throughout embryonic development. SK1 and SK2 transcripts are expressed as early as E11, when the neural tube is closed and the three vesicles (prosencephalon, mesencephalon, and rhombencephalon) are present (Fig. 2A,B). Conversely, SK3 expression is below the detection limit at E11 (Fig. 2C). At E12, a clearer pattern of expression emerges for SK2, which shows the strongest signals in the telencephalic and rhombencephalic regions (Fig. 2E), whereas the expression of SK1 mRNA is partially overlapping but more restricted (Fig. 2D) and SK3 is still barely detectable (Fig. 2F). By E13, a clear increase in the levels of SK1 (Fig. 2G) and SK2 (Fig. 2H) transcripts can be observed, but SK3 is still weak (Fig. 2I). Both SK1 and SK2 transcripts are present in the primordial cortex, hippocampus, pons, and spinal cord. At E15 (Fig. 2J–L) and E17 (Fig. 2M–O), specific brain regions are better defined, and the distribution of the SK1 and SK2 transcripts overlaps in several but not in all brain regions, whereas SK3 presents a clearly distinct distribution pattern. At these stages, SK1 transcripts are strongly expressed in the differentiating striatum, trigeminal ganglion, spinal cord, pons, and cerebellum, and weaker signals are detectable in the cortex and hippocampus (Fig. 2J,M, Table1). SK2 mRNA presents a similar distribution but at different levels, with cortex, superior colliculus, and spinal cord showing the strongest expression and the striatum weaker signals (Fig. 2K,N, Table2). Expression of SK3 is detectable in the cortex, pons, and spinal cord (Fig. 2L,O, Table3).

**Table 2:** Prenatal Distribution (E15–E21) of the Small-Conductance Calcium-Activated Potassium Channel Subunit SK2^1^

Brain region	E15	E17	E19	E21
**Olfactory system**				
Olfactory bulb				
Glomerular layer	np	np	−	−
Mitral cell layer	np	np	+	+
Internal granular layer	np	np	np	+
Accessory olfactory bulb	np	−	−	−
Anterior olfactory nucleus	np	•	++	++
Olfactory tubercle	np	np	+	++
**Cerebral cortex**				
Piriform cortex	•	•	++	++
Primordial plexiform layer	+++	np	np	np
Cortical neuroepithelium	++	+	++	+
Molecular layer	np	−	−	−
Cortical plate	np	+++	+++	+++
Intermediate zone	np	+	++	+++
Subventricular zone	np	+	+	−
Hippocampal formation				
Subicular neuroepithelium	++	++	np	np
Subiculum (or diff. field)	np	+++	+++	+++
Hippocampal neuroepithelium	++	++	np	np
Hippocampus (or diff. field)	+++	+++	np	np
CA1	np	np	+++	+++
CA3	np	np	++	++
Dentate gyrus granule cells	np	np	−	−
**Basal ganglia**				
Striatum (or diff. field)	+++	++	−	−
**Thalamus**				
Anterior thalamus (or diff. field)	−	++	++	++
Posterior thalamus (or diff. field)	−	++	++	++
Intermediate thalamus (or diff. field)	−	•	++	++
**Trigeminal ganglion**	++	+++	+++	+++
**Inferior glossopharyngeal ganglion**	•	•	+++	+++
**Pons (or diff. field)**	+++	++	+++	•
**Cerebellum**				
Cerebellar neuroepithelium	−	−	np	np
Cerebellum (or diff. field)	+++	+++	np	np
Deep nuclei	np	np	−	−
Purkinje cell layer	np	np	+++	+++
External germinal layer	np	•	−	−
Granule cell layer	np	np	−	−
**Spinal cord**	+++	+++	•	•
**Dorsal root ganglia**	++	+++	•	•
**Intestine**	+++	++	•	•

1 +++, strong; ++, moderate; +, weak; −, signals very low or below the threshold limit of detection; np, not present; •, not clearly identifiable; Diff. field, differentiating field.

**Table 3:** Prenatal Distribution (E15–E21) of the Small-Conductance Calcium-Activated Potassium Channel Subunit SK3^1^

Brain region	E15	E17	E19	E21
**Olfactory system**				
Olfactory bulb				
Glomerular layer	np	np	++	+
Mitral cell layer	np	np	−	−
Internal granular layer	np	np	np	−
Accessory olfactory bulb	np	−	+++	++
Anterior olfactory nucleus	np	•	+++	++
Olfactory tubercle	np	np	+	+
**Cerebral cortex**				
Piriform cortex	•	•	+	++
Primordial plexiform layer	+++	np	np	np
Cortical neuroepithelium	+	+	+	+
Molecular layer	np	−	−	−
Cortical plate	np	+++	−	−
Intermediate zone	np	+++	+	+
Subventricular zone	np	+++	+++	++
Hippocampal formation				
Subicular neuroepithelium	+	++	np	np
Subiculum (or diff. field)	np	+++	++	++
Hippocampal neuroepithelium	+	+	np	np
Hippocampus (or diff. field)	+++	++	np	np
CA1	np	np	+++	++
CA3	np	np	+	+
Dentate gyrus granule cells	np	np	+	+
**Basal ganglia**				
Striatum (or diff. field)	−	−	−	−
**Thalamus**				
Anterior thalamus (or diff. field)	+	+++	+++	++
Posterior thalamus (or diff. field)	−	++	++	++
Intermediate thalamus (or diff. field)	+++	+++	+++	++
**Trigeminal ganglion**	+++	++	−	−
**Inferior glossopharyngeal ganglion**	•	•	−	−
**Pons (or diff. field)**	+	+++	++	•
**Cerebellum**				
Cerebellar neuroepithelium	−	−	np	np
Cerebellum (or diff. field)	−	−	np	np
Deep nuclei	np	np	−	−
Purkinje cell layer	np	np	−	−
External germinal layer	np	•	−	−
Granule cell layer	np	np	−	−
**Spinal cord**	+++	+++	•	•
**Dorsal root ganglia**	++	++	•	•
**Intestine**	+++	+++	•	•

1 +++, strong; ++, moderate; +, weak; −, signals very low or below the threshold limit of detection; np, not present; •, not clearly identifiable; Diff. field, differentiating field.

Analysis of sections through rat embryos from E11 to E17 showed that early expression of all subunits is not restricted to the nervous system. The embryonic heart expresses the three subunit mRNAs, with clear signals at E15–E17 (Fig. 2J–O). At E17, expression of SK3 (Fig. 2O), but not of SK1 or SK2, was observed in the metanephron. SK1 and SK2 are expressed in vertebral cartilage at E15 and E17 (Fig. 2J,K,M,N). Finally, SK2 (Fig. 2K) and SK3 (Fig. 2O) are visible in the intestine wall starting at E15 (Tables2, 3).

At later stages of brain development (E19, E21; Fig. 3) SK1 and SK2 transcripts still show overlapping patterns, although their relative levels of expression vary in different brain regions, with strong signals for both subunit transcripts in the cortex and hippocampus (Fig. 3A,B,D,E, Tables1, 2). SK2 displays a strong expression in developing Purkinje cells (Fig. 2B,D, Table2). The expression of SK3 in the cortex and hippocampus changes dramatically, decreasing from E17 (Fig. 2O) to E21 (Fig. 3C,F, Table3).

### Embryonic distribution of SK1, SK2, and SK3 transcripts in the spinal cord and peripheral nervous system

Whole-embryo hybridizations reveal SK channel subunit expression in the developing spinal cord, with a diffuse expression of SK1, SK2, and SK3 in both dorsal and ventral horns (Fig. 4, Tables3). While the overall expression level of SK1 increases from E15 to E17 (Fig. 4A,C, Table1), the level of SK2 is relatively stable at these developmental stages (Fig. 4E,G, Table2), and SK3 expression increases especially in the ventral horn and is very pronounced in the ventral commissure (Fig. 4I,K, Table3).

All three SK channel subunits are expressed in sensory neurons of the dorsal root ganglia (DRG; Figs. 2M–O, 4). SK1 (Fig. 4B,D) and SK2 (Fig. 4F,H) show a clear increase, reaching strong expression at E17 in DRG neurons (Tables1, 2), but SK3 displays a more diffuse pattern, with an increase between E15 and E17 and labeling also of the proximal part of the spinal nerves extending from the DRG (Fig. 4J,L, Table3). Together with the intense labeling of the ventral commissure in the spinal cord, the SK3 signal in spinal nerves suggests active axonal transport of SK3 mRNAs.

In the cranial sensory ganglia, strong signals for SK1 and SK2 (Tables1, 2), but not for SK3 (Table3), were observed at E19 (Fig. 13A–C) and E21 (Fig. 13J,K left) in the inferior ganglion of the glossopharyngeal nerve, innervating the pharynx, tonsils, tongue, middle ear, auditory tube, and ear canal. In the trigeminal ganglion, all three SK subunits were observed at E15 (Fig. 12J–L) and E17 (Fig. 2M–O, Tables3). SK1 displayed strong expression (Fig. 2J,M). At E19 and E21, SK1 expression persists (Fig. 13A, Table1) and SK2 levels increase (Fig. 13B, Table2), but SK3 shows a pronounced decline in expression (Fig. 3C, Table1).

### Embryonic and postnatal distribution of SK1, SK2, and SK3 transcripts in specific brain regions

**Figure 10:**
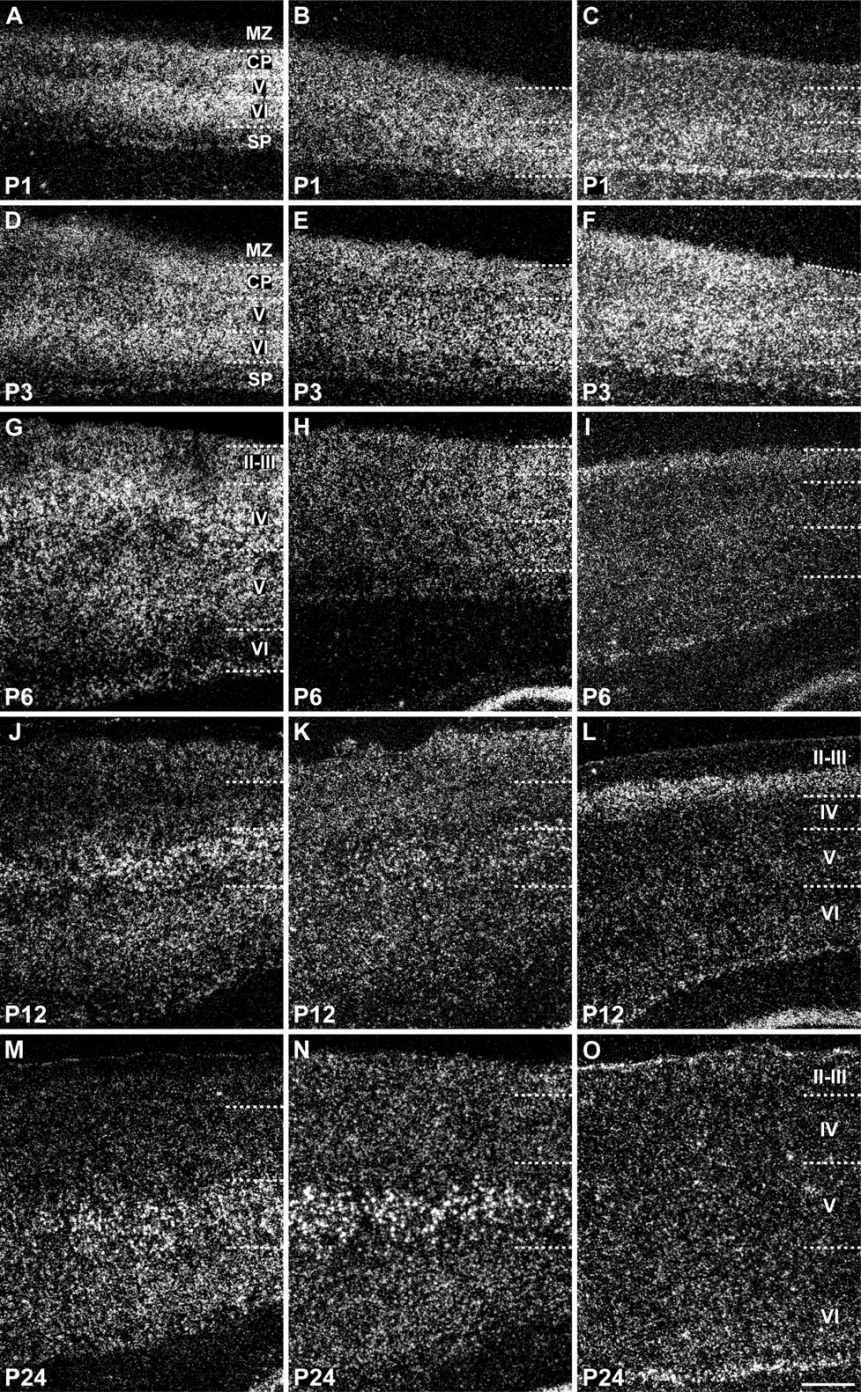
Laminar distribution of SK1, SK2, and SK3 transcripts in the neocortex during postnatal development. SK1 (A,D,G,J,M) and SK2 (B,E,H,K,N) display a similar developmental pattern, starting with relatively strong expression in multiple layers at P1–P3 and leading to signals that are predominant in layer V (V) neurons at P24, but SK3 (C,F,I,L,O) shows a progressive decline in expression in all layers, with a moderate level persisting in the deep part of layer VI (VI) at P24. For abbreviations see list. For details on the distribution see Tables6. Scale bar = 400 μm.

To analyze the distribution patterns of SK transcripts after birth, we extended our in situ hybridization analysis and mapped the distributions of SK1, SK2, and SK3 mRNAs in the rat postnatal nervous system between P1 and P24 by examination of X-ray film images (Figs. 7) and emulsion-coated sections (Figs. 10, 12, 14, 15). The results of the evaluation at the cellular level are summarized in Tables6.

**Figure 11:**
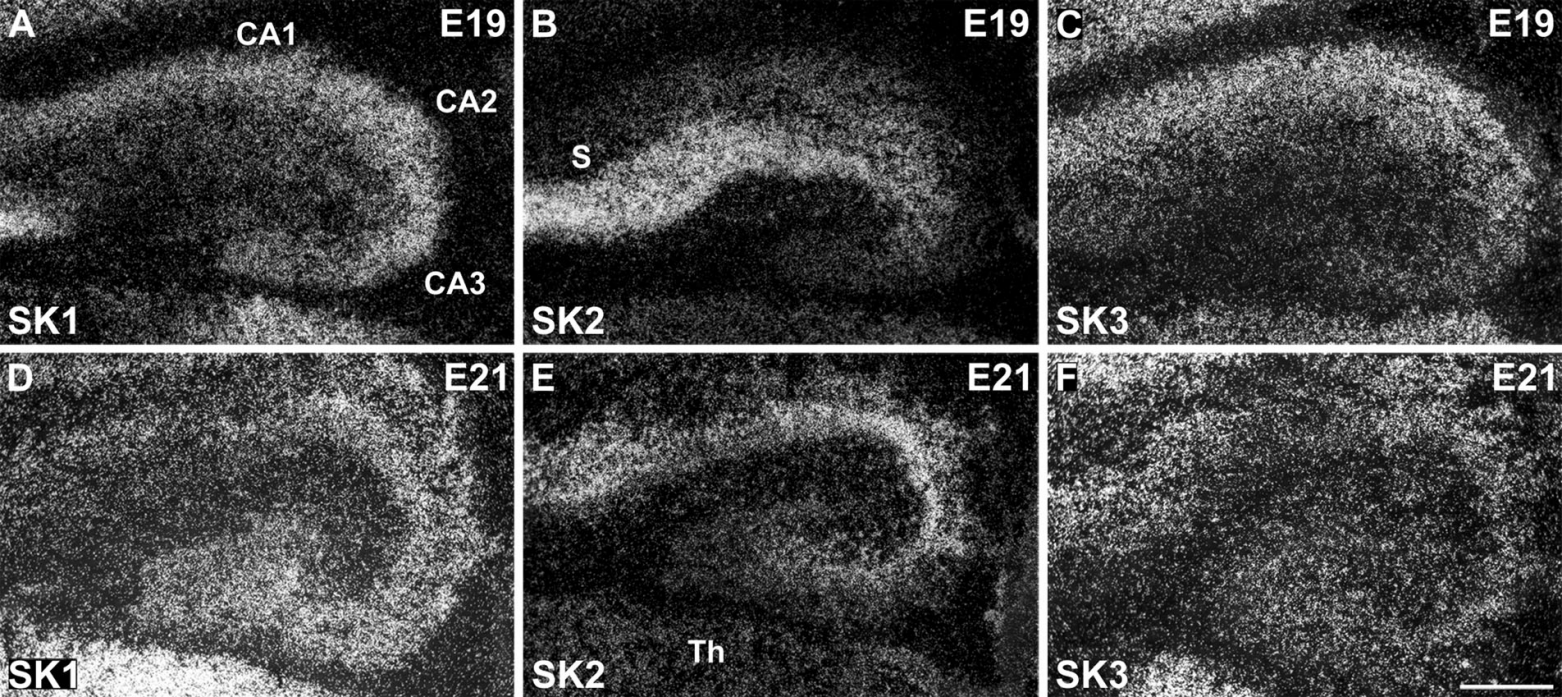
All three SK channel transcripts are expressed in the hippocampal formation at E19 and E21. SK2 displays the strongest expression in the subiculum (S) and CA1 layer (B,E). SK1 (A,D) and SK2 (B,E) subunits display overlapping expression patterns in the differentiating CA1 and CA3 layers, and SK3 is expressed overall at lower levels in all hippocampal fields at these developmental stages (C,F). For abbreviations see list. For details on the distribution see Tables3. Scale bar = 250 μm.

**Figure 12:**
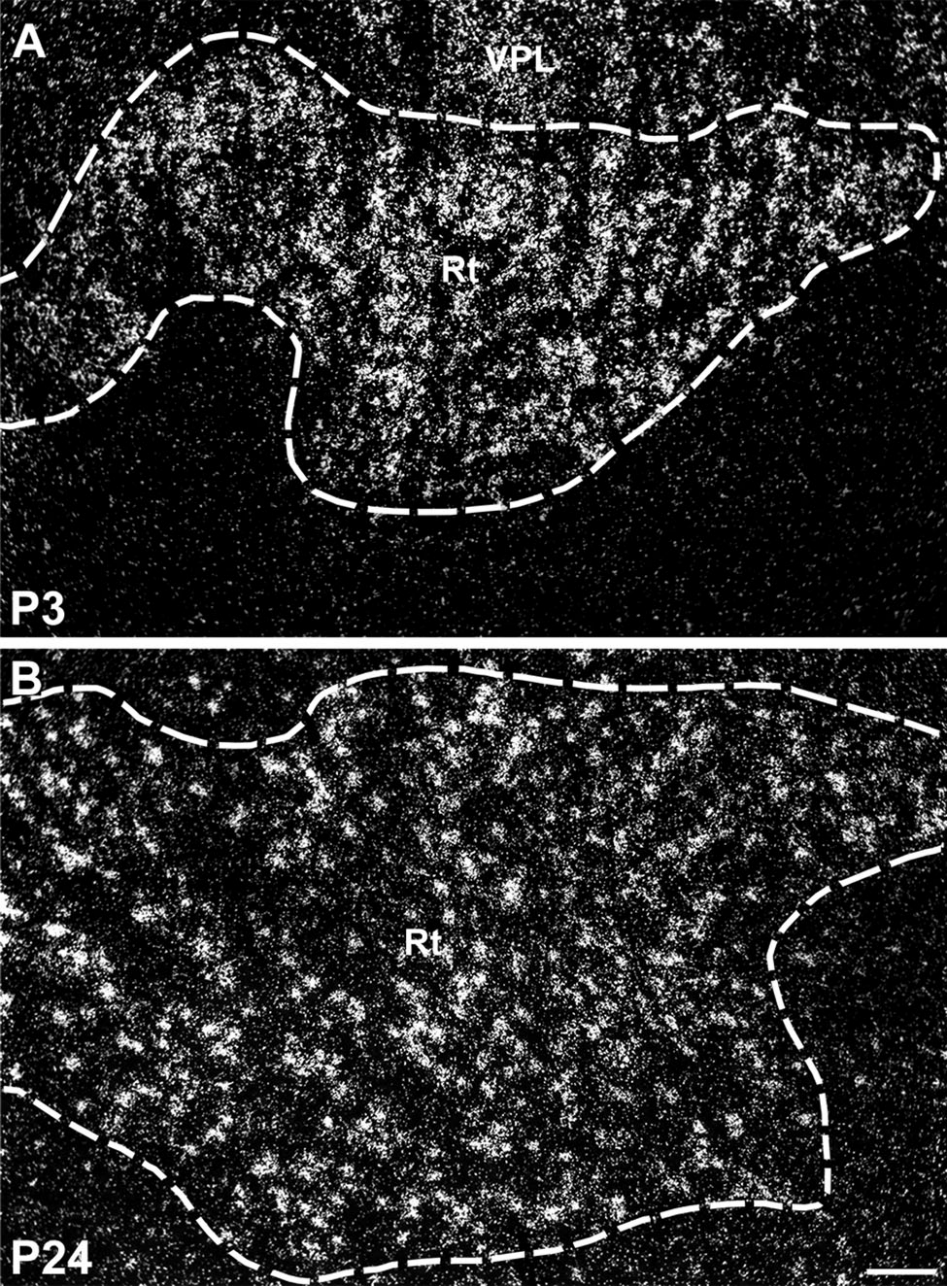
Darkfield photomicrographs showing strong expression of SK2 transcripts in the reticular thalamic nucleus (Rt) at P3 (A) and P24 (B). For abbreviations see list. Scale bar = 100 μm.

**Figure 13:**
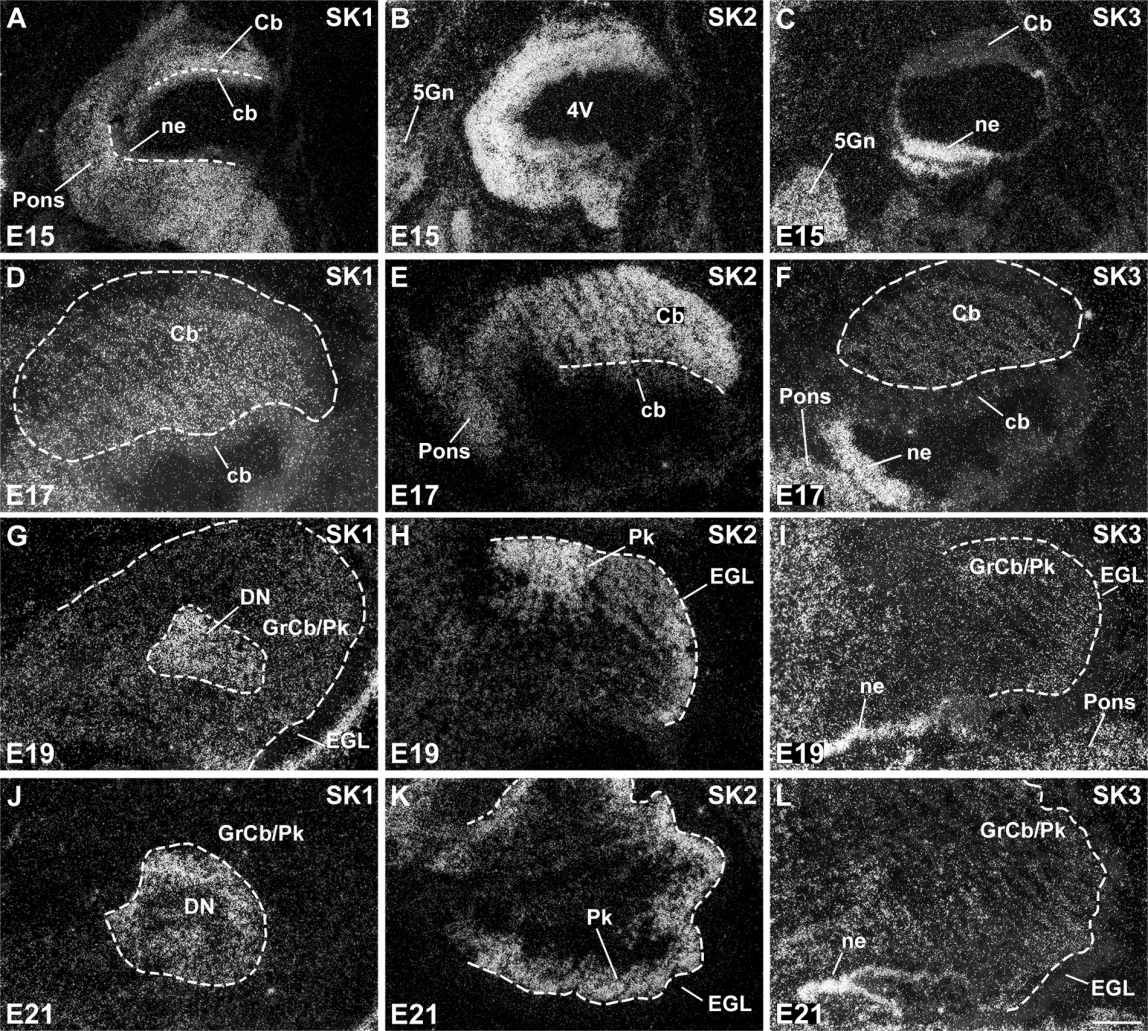
Expression of SK1, SK2, and SK3 in the embryonic differentiating cerebellum and precerebellar nuclei. SK1 displays a diffuse expression pattern in the cerebellar differentiating field (Cb) at E15 (A) and E17 (D), with strong expression predominantly in the deep cerebellar nuclei (DN) at E19 (G) and E21 (J). SK2 is expressed more strongly than SK1 in the cerebellar differentiating field at E15 (B) and E17 (E). Its strongest expression is subsequently observed in the differentiating Purkinje cell layer (Pk) at E19 (H) and E21 (K). SK3 shows an overall weak and diffuse expression in the cerebellar differentiating field from E15 (C) to E21 (L), in contrast to strong expression in the precerebellar (C,F) and cochlear (I,L) neuroepithelia. For abbreviations see list. For details on the distribution see Tables3. Scale bars = 400 μm in L (applies to D–J,L); 800 μm for A,B,C,K.

**Figure 14:**
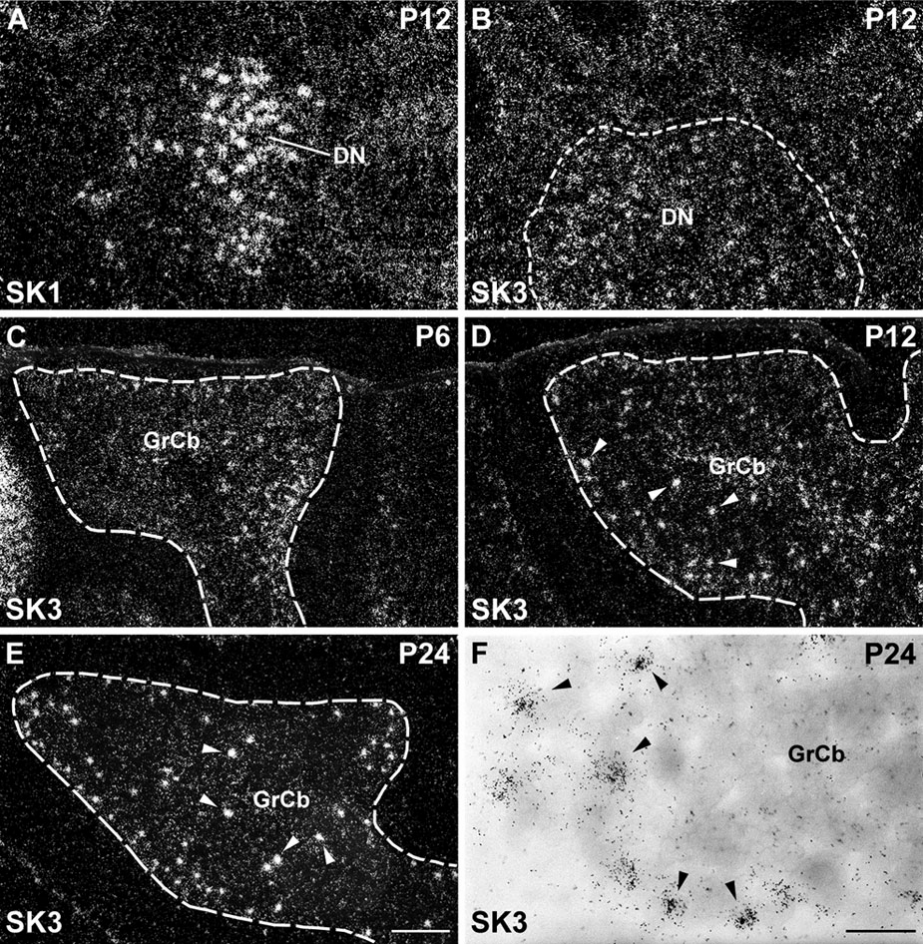
Expression of SK1 and SK3 subunits in the postnatal cerebellum. SK1 shows strong expression in large neurons of the deep cerebellar nuclei (DN) at P12 (A). SK3 is also expressed at moderate levels in the deep nuclei at P12 (B). C–E: Darkfield photomicrographs displaying strong signal for the expression of SK3 transcripts in scattered Golgi cells at P6 (C), P12 (D), and P24 (E). Golgi cells are depicted at higher magnification in F, using brightfield optics that reveal clear clusters of silver grains on scattered cell nuclei (arrowheads). For abbreviations see list. For details on the distribution see Tables4 and 6. Scale bars = 300 μm in E (applies to A–E); 40 μm in F.

**Figure 15:**
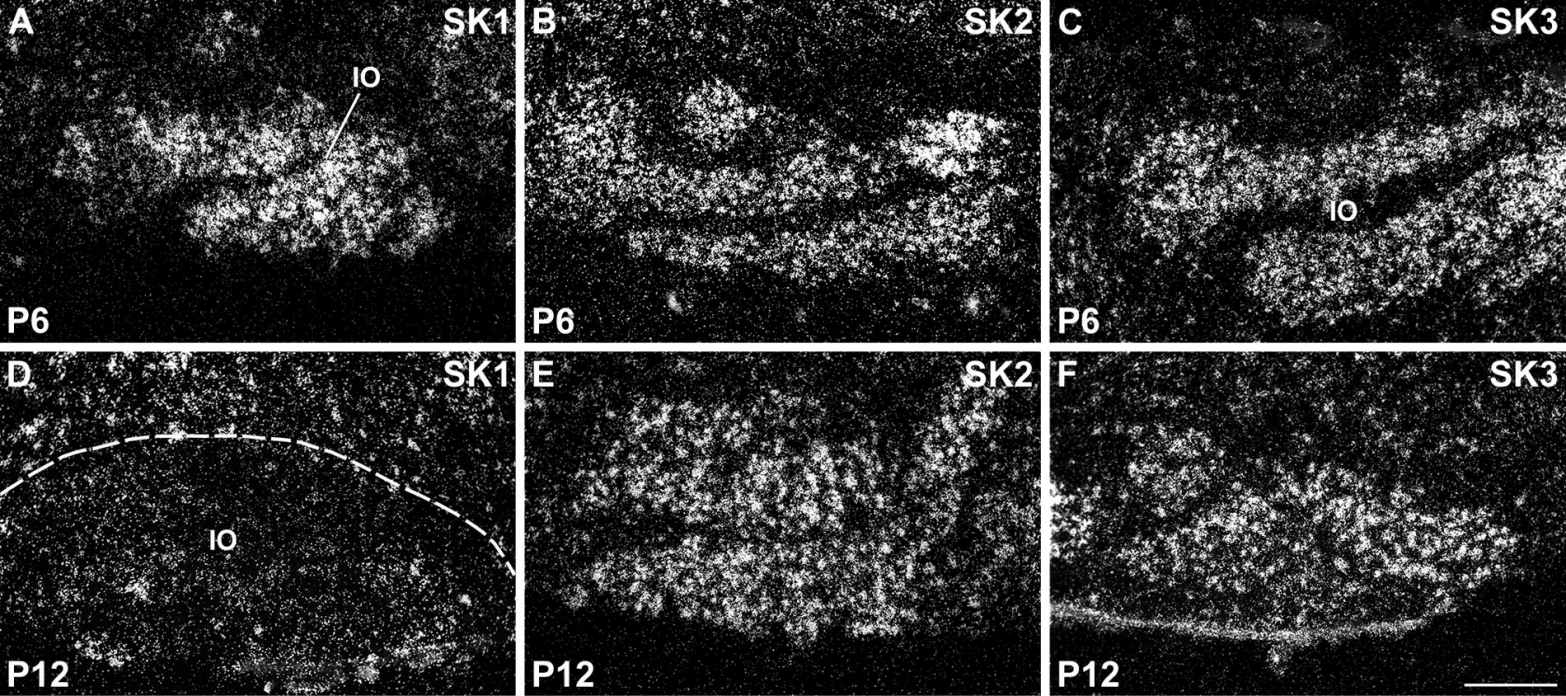
Expression of SK channel subunits in the inferior olivary nucleus (IO). SK1 shows a strong hybridization signal in inferior olivary neurons up to P6 (A), which clearly declines at P12 (D). Conversely, both SK2 (B,E) and SK3 (C,F) display strong expression throughout postnatal development. For details on the distribution see Tables6. Scale bar = 250 μm.

**Table 4:** Postnatal Distribution (P1–P24) of the Small-Conductance Calcium-Activated Potassium Channel Subunit SK1^1^

Brain region	P1	P3	P6	P12	P24
**Olfactory system**					
Endopiriform nucleus	+	+	++	+++	++
Olfactory bulb					
Glomerular layer	−	−	+	+	−
Mitral cell layer	−	−	+	++	++
Int. granular layer	+	+	++	++	++
Accessory olfactory bulb	−	−	−	−	−
Anterior olfactory nucleus	+++	+++	+++	+++	+++
Olfactory tubercle	+++	+++	+++	+++	+++
Islands of Calleja	+++	+++	+++	+++	+++
**Cerebral cortex**					
Piriform cortex	+++	+++	+++	+++	++
Entorhinal cortex	+++	+++	+++	+++	+++
Marginal zone	−	+	np	np	np
Cortical plate	+++	+++	np	np	np
Subplate	+	+	np	np	np
Alternative layer II/III	np	np	++	+	+
Alternative layer IV	np	np	+++	+	+
Alternative layer V	+++	+++	+++	+++	+++
Alternative layer VI	+++	+++	++	++	++
Hippocampal formation					
Subiculum	+++	+++	+++	+++	+++
CA1	++	+++	+++	+++	+++
CA2/CA3	++	++	++	+++	++
Dentate gyrus granule cells	+	+	+	+	+
Tenia tecta	+++	+++	+++	+++	+++
**Basal nuclei**					
Caudate putamen	++	++	++	++	++
Globus pallidus	+	+	+	+	+
Entopeduncular nucleus	+++	+++	+++	+++	+++
Nucleus accumbens	++	++	++	+	+
Claustrum	+	+	+	++	++
Substantia nigra					
Pars reticulata	+++	+++	+++	+++	+
Pars compacta	−	−	−	−	−
**Septum**					
Septum	++	++	++	++	+
Bed nucleus stria terminalis	−	−	−	−	−
**Amygdala**					
Anterior amygdaloid area	++	++	++	++	++
Anterior cortical amygdaloid nucleus	−	−	−	−	−
Medial amygdaloid nucleus	++	++	++	++	++
Posterior cortical amygdaloid nucleus	+++	+++	++	++	−
Basolateral amygdaloid nucleus	−	−	++	+++	+++
Basomedial amygdaloid nucleus	−	−	+	+	+
Amygdalohippocampal area	+	+	++	+++	+++
Amygdalopiriform transition	−	−	−	−	−
Central amygdaloid nucleus	−	−	−	−	−
**Thalamus**					
Reticular thalamic nucleus	+++	+++	+++	+++	+++
Anterodorsal nucleus	+++	+++	+++	+++	+++
Anteroventral nucleus	+++	+++	+++	+++	+++
Anteromedial nucleus	−	−	−	−	−
Laterodorsal nucleus	−	−	−	−	−
Paratenial nucleus	•	•	++	++	++
Mediodorsal nucleus	+++	+++	+++	+++	+++
Ventrolateral nucleus	+++	+++	+++	+++	+
Paraventricular nucleus, anterior	•	•	•	+++	+++
Paraventricular nucleus, posterior	•	•	•	+++	+++
Central medial nucleus	+++	+++	+++	−	−
Ventromedial nucleus	•	•	+++	+++	+
Ventroposterolateral thalamic nucleus	•	•	+++	+++	+
Ventroposteromedial thalamic nucleus	•	•	+++	+++	+
Posterior thalamic nucleus	•	•	+++	++	+
Centrolateral nucleus	•	•	+++	+++	+
Parafascicular nucleus	++	++	+++	+++	+++
Lateral geniculate nucleus, dorsal	•	•	+++	+++	−
Lateral geniculate nucleus, ventral	•	•	+	++	+
Medial geniculate nucleus	•	•	+++	+++	−
Lateroposterior nucleus	+++	+++	++	+	−
**Habenula**	−	−	−	−	−
**Hypothalamus**					
Preoptic nuclei	++	++	+	+	++
Magnocellular preoptic nucleus	•	•	++	++	++
Anterior hypothalamic area	++	++	−	−	−
Lateral hypothalamic area	•	•	−	−	+
Arcuate nucleus	−	−	−	−	−
Ventromedial hypothalamic nucleus	++	++	+	−	−
Dorsomedial hypothalamic nucleus	−	−	−	−	−
Tuberomammillary nucleus	•	•	+++	+++	+++
Premammillary nucleus	−	−	−	−	−
Medial mammillary nucleus	++	++	++	++	++
Lateral mammillary nucleus	+	+	+	+	+
Magnocellular nucleus of lateral hypothalamus	•	•	+++	+++	+++
Zona incerta	+++	+++	+++	+++	+++
**Brainstem**					
**Reticular formation and related tegmental nuclei**					
Medullary reticular formation	•	•	+++	+++	+++
Gigantocellular reticular nucleus	+++	+++	+++	+++	+++
Paragigantocellular reticular n.	•	•	++	++	++
Intermediate reticular nucleus	•	•	+++	+++	+++
Parvocellular reticular nucleus	++	++	+++	+++	+++
Pontine reticular formation	++	+++	+++	+++	++
Dorsomedial tegmental field	•	•	−	−	−
Laterodorsal tegmental nuclei	•	•	+++	+++	+++
Dorsal tegmental nucleus	−	−	−	−	−
**Monoaminergic systems**					
Dorsal raphe nucleus	−	−	−	−	−
Locus coeruleus	−	−	−	−	−
Ventral tegmental area	−	−	−	−	−
**Nuclei associated with respiratory, cardiovascular and other autonomic functions**					
Nucleus of the solitary tract	++	++	++	+++	+++
Parabrachial nucleus	−	−	−	−	−
Dorsal motor nucleus of vagus	−	−	−	−	−
Ambiguus nucleus	•	•	+++	+++	+++
**Orofacial motor nuclei**					
Motor trigeminal nucleus	++	++	+++	+++	+++
Facial nucleus	++	++	+++	+++	+++
Hypoglossal nucleus	++	++	++	+++	+++
**Nuclei belonging to the somatosensory system**					
Gracile/cuneate nucleus	+++	+++	+++	+++	+++
Principal sensory trigeminal nucleus	+	++	+++	+++	+++
Spinal trigeminal nucleus	+++	+++	+++	+++	+++
**Nuclei belonging to the auditory system**					
Cochlear nuclei	+++	+++	+++	+++	+++
Lateral superior olive	+++	+++	+++	+++	++
Nucleus trapezoid body	−	−	+++	+++	+++
Nuclei of the lateral lemniscus	++	+++	+++	+++	+++
Inferior colliculus	+++	+++	+++	+++	+
**Nuclei belonging to the visual system**					
Superior colliculus	++	++	+	+	+
Parabigeminal nucleus	+++	+++	+++	+++	+++
**Vestibular and precerebellar nuclei**					
Vestibular nuclei	+++	+++	+++	+++	+++
Pontine nuclei	++	++	+++	+++	+++
Inferior olivary nucleus	+++	+++	+++	−	−
Red nucleus	+++	+++	+++	+++	+++
**Cerebellum**					
Deep nuclei	+++	+++	+++	+++	+++
Purkinje cells	−	−	−	−	−
Golgi cells	−	−	−	−	−
Granule cells	−	+	+	+	+

1 +++, strong; ++, moderate; +, weak; −, signals very low or below the threshold limit of detection; np, not present; •, not clearly identifiable.

#### Cerebral cortex

The cerebral cortex, the largest structure in the mammalian telencephalon, includes the neocortex and limbic cortex, the piriform cortex, and the hippocampal region (Bayer and Altman, 1995). The three SK channel subunits display different ontogenetic changes during the embryonic development of the neocortex. SK1 (Fig. 2G, Table1) and SK2 (Fig. 2H, Table2) are clearly expressed in the differentiating cortex at E13, but SK3 is barely detectable at this developmental stage (Fig. 2I, Table3).

SK2 transcripts are present in the cortical neuroepithelium at E15, with strong expression in the thin primordial plexiform layer of differentiating neurons, likely formed by Cajal-Retzius and subplate neurons (Fig. 8E,F, Table2). SK2 is strongly expressed in layer V neurons during postnatal development (P12 and P24; Fig. 10K,N, Table5) and is present in both layer V and layer VI neurons in the adult neocortex (Stocker and Pedarzani, 2000). Layer VI and V neurons have largely reached the cortical plate by E17–E18 (Bayer and Altman, 1995), when SK2 displays maximal expression levels in the cortical plate (Fig. 8G,H). The strong signals observed in the cortical plate at E17 suggest early expression of SK2 in layer V–VI neurons. Expression of SK2 mRNA is still strong in the cortical plate and moderate in the intermediate zone at E19 (Fig. 9B) and strong in both cortical plate and intermediate zone at E21 (Fig. 9E, Table2).

**Table 5:** Postnatal Distribution (P1–P24) of the Small-Conductance Calcium-Activated Potassium Channel Subunit SK2^1^

Brain region	P1	P3	P6	P12	P24
**Olfactory system**					
Endopiriform nucleus	++	++	++	++	++
Olfactory bulb					
Glomerular layer	−	−	−	+	−
Mitral cell layer	+	+	++	++	++
Int. granular layer	+	+	++	++	++
Accessory olfactory bulb	−	−	−	−	−
Anterior olfactory nucleus	++	++	++	+++	+++
Olfactory tubercle	++	++	+	++	++
Islands of Calleja	++	++	++	++	++
**Cerebral cortex**					
Piriform cortex	++	++	++	+++	++
Entorhinal cortex	+++	+++	++	+++	+++
Marginal zone	−	−	np	np	np
Cortical plate	+++	+++	np	np	np
Subplate	+	++	np	np	np
Alternative layer II/III	np	np	++	+	+
Alternative layer IV	np	np	+++	+	+
Alternative layer V	+++	+++	++	+++	+++
Alternative layer VI	+++	+++	++	++	+++
Hippocampal formation					
Subiculum	+++	+++	+++	+++	+++
CA1	++	++	+++	+++	+++
CA2/CA3	++	++	++	+++	+++
Dentate gyrus granule cells	+	+	+	+	+
Tenia tecta	+++	+++	+++	+++	+++
**Basal nuclei**					
Caudate putamen	+	+	+	+	+
Globus pallidus	−	−	−	−	−
Entopeduncular nucleus	++	++	++	++	++
Nucleus accumbens	−	−	−	−	−
Claustrum	−	−	−	+	+
Substantia nigra					
Pars reticolata	−	+	++	++	++
Pars compacta	−	−	−	−	−
**Septum**					
Septum	++	++	++	++	++
Bed nucleus stria terminalis	+	+	+	+	+
**Amygdala**					
Anterior amygdaloid area	++	++	++	++	++
Anterior cortical amygdaloid nucleus	++	++	++	++	++
Medial amygdaloid nucleus	+	+	++	++	++
Posterior cortical amygdaloid nucleus	++	++	++	++	++
Basolateral amygdaloid nucleus	+++	+++	+++	+++	+++
Basomedial amygdaloid nucleus	+	+	++	+++	+++
Amygdalohippocampal area	+++	+++	+++	+++	+++
Amygdalopiriform transition	+	++	++	++	++
Central amygdaloid nucleus	++	++	++	++	++
**Thalamus**					
Reticular thalamic nucleus	+++	+++	+++	+++	+++
Anterodorsal nucleus	++	+++	+++	+++	+++
Anteroventral nucleus	++	++	++	++	++
Anteromedial nucleus	+	++	++	++	++
Laterodorsal nucleus	+	−	−	+	++
Paratenial nucleus	•	•	+	+	+
Mediodorsal nucleus	++	++	++	++	++
Ventrolateral nucleus	+++	++	++	++	++
Paraventricular nucleus, anterior	•	•	•	−	−
Paraventricular nucleus, posterior	•	•	•	+	+
Central medial nucleus	−	−	−	+	+
Ventromedial nucleus	•	•	++	++	++
Ventroposterolateral thalamic nucleus	•	•	+++	+++	+++
Ventroposteromedial thalamic nucleus	•	•	+++	+++	+++
Posterior thalamic nucleus	•	•	+	+	++
Centrolateral nucleus	•	•	++	++	++
Parafascicular nucleus	++	++	+++	+++	++
Lateral geniculate nucleus, dorsal	•	•	++	+	+
Lateral geniculate nucleus, ventral	•	•	+	+	++
Medial geniculate nucleus	•	•	++	++	++
Lateroposterior nucleus	−	−	+	++	++
**Habenula**	++	++	++	++	++
**Hypothalamus**					
Preoptic nuclei	++	++	+	+	++
Magnocellular preoptic nucleus	•	•	++	++	++
Anterior hypothalamic area	−	−	−	−	−
Lateral hypothalamic area	•	•	++	++	++
Arcuate nucleus	−	−	−	−	−
Ventromedial hypothalamic nucleus	+	+	+	+	+
Dorsomedial hypothalamic nucleus	+	+	+	+	+
Tuberomammillary nucleus	•	•	++	++	++
Premammillary nucleus	+	+	+	+	+
Medial mammillary nucleus	++	++	++	++	++
Lateral mammillary nucleus	−	++	++	++	++
Magnocellular nucleus of lateral hypothalamus	•	•	−	−	−
Zona incerta	++	++	++	++	++
**Brainstem**					
**Reticular formation and related tegmental nuclei**					
Medullary reticular formation	•	•	++	+++	+++
Gigantocellular reticular nucleus	++	+++	+++	+++	+++
Paragigantocellular reticular nucleus	•	•	++	+++	+++
Intermediate reticular nucleus	•	•	++	++	++
Parvocellular reticular nucleus	++	++	++	++	++
Pontine reticular formation	+++	+++	+++	+++	+++
Dorsomedial tegmental field	•	•	+	+	++
Laterodorsal tegmental nuclei	•	•	++	++	++
Dorsal tegmental nucleus	−	−	+	++	++
**Monoaminergic systems**					
Dorsal raphe nucleus	−	−	−	++	++
Locus coeruleus	−	−	−	−	−
Ventral tegmental area	−	−	−	−	−
**Nuclei associated with respiratory, cardiovascular, and other autonomic functions**					
Nucleus of the solitary tract	+	+	+	+	+
Parabrachial nucleus	−	−	++	++	++
Dorsal motor nucleus of vagus	−	−	−	−	−
Ambiguus nucleus	•	•	+++	+++	+++
**Orofacial motor nuclei**					
Motor trigeminal nucleus	+++	+++	+++	+++	+++
Facial nucleus	+	+++	+++	+++	+++
Hypoglossal nucleus	++	+++	+++	+++	+++
**Nuclei belonging to the somatosensory system**					
Gracile/cuneate nucleus	+++	+++	+++	+++	+++
Principal sensory trigeminal nucleus	+++	+++	+++	+++	+++
Spinal trigeminal nucleus	+++	+++	+++	+++	+++
**Nuclei belonging to the auditory system**					
Cochlear nuclei	+++	+++	+++	+++	+++
Lateral superior olive	++	++	+++	+++	+++
Nucleus trapezoid body	+++	+++	+++	+++	+++
Nuclei of the lateral lemniscus	++	++	+++	+++	+++
Inferior colliculus	+++	+++	++	++	++
**Nuclei belonging to the visual system**					
Superior colliculus	++	++	++	++	++
Parabigeminal nucleus	++	++	++	+++	+++
**Vestibular and precerebellar nuclei**					
Vestibular nuclei	+++	+++	+++	+++	+++
Pontine nuclei	+++	+++	+++	+++	+++
Inferior olivary nucleus	+++	+++	+++	+++	+++
Red nucleus	++	++	++	++	++
**Cerebellum**					
Deep nuclei	++	++	++	+++	+++
Purkinje cells	+++	+++	+++	+++	++
Golgi cells	−	−	−	−	−
Granule cells	−	−	+	++	++

1 +++, strong; ++, moderate; +, weak; −, signals very low or below the threshold limit of detection; np, not present; •, not clearly identifiable.

**Table 6:** Postnatal Distribution (P1–P24) of the Small-Conductance Calcium-Activated Potassium Channel Subunit SK3^1^

Brain region	P1	P3	P6	P12	P24
**Olfactory system**					
Endopiriform nucleus	++	+++	++	+++	+++
Olfactory bulb					
Glomerular layer	++	++	++	++	++
Mitral cell layer	++	+	+	+	++
Int. granular layer	++	++	++	++	++
Accessory olfactory bulb	+++	+++	+++	+++	+++
Anterior olfactory nucleus	+++	+++	+++	+++	+++
Olfactory tubercle	++	+++	+++	+++	++
Islands of Calleja	++	++	++	++	++
**Cerebral cortex**					
Piriform cortex	+++	+++	+++	++	++
Entorhinal cortex	+++	+++	++	+++	+++
Marginal zone	++	−	np	np	np
Cortical plate	++	+++	np	np	np
Subplate	++	+++	np	np	np
Alternative layer II/III	np	np	++	+++	+
Alternative layer IV	np	np	++	+	+
Alternative layer V	+++	+++	++	+	+
Alternative layer VI	+++	+++	++	+	++
Hippocampal formation					
Subiculum	++	++	+++	+++	+++
CA1	−	+	++	+++	−
CA2/CA3	−	−	+	++	+
Dentate gyrus granule cells	−	−	+++	+++	+++
Tenia tecta	+++	+++	+++	++	++
**Basal nuclei**					
Caudate putamen	+++	+++	+++	+++	+++
Globus pallidus	++	++	++	++	++
Entopeduncular nucleus	−	−	−	−	−
Nucleus accumbens	+++	+++	+++	+++	+++
Claustrum	−	−	+	++	++
Substantia nigra					
Pars reticulata	++	++	−	−	−
Pars compacta	+++	+++	+++	+++	+++
**Septum**					
Septum	+++	+++	+++	+++	+++
Bed nucleus stria terminalis	+++	+++	+++	+++	+++
**Amygdala**					
Anterior amygdaloid area	++	++	++	++	++
Anterior cortical amygdaloid nucleus	+++	+++	+++	++	+++
Medial amygdaloid nucleus	+++	+++	+++	+++	+++
Posterior cortical amygdaloid nucleus	+++	+++	+++	+++	+++
Basolateral amygdaloid nucleus	+++	+++	+++	+++	+++
Basomedial amygdaloid nucleus	+++	+++	+++	++	+++
Amygdalohippocampal area	+++	+++	+++	+++	+++
Amygdalopiriform transition	+++	+++	++	++	++
Central amygdaloid nucleus	+++	+++	+++	+++	+++
**Thalamus**					
Reticular thalamic nucleus	−	−	−	−	−
Anterodorsal nucleus	+++	+++	+++	+++	+++
Anteroventral nucleus	+	+	+	+	+++
Anteromedial nucleus	+++	+++	++	+++	+++
Laterodorsal nucleus	+++	+++	+++	+++	+++
Paratenial nucleus	•	•	++	++	+++
Mediodorsal nucleus	++	++	++	++	+++
Ventrolateral nucleus	++	++	++	++	+++
Paraventricular nucleus, anterior	•	•	+++	++	+++
Paraventricular nucleus, posterior	•	•	++	++	++
Central medial nucleus	−	−	++	++	+++
Ventromedial nucleus	•	•	++	++	++
Ventroposterolateral thalamic nucleus	•	•	+++	+++	++
Ventroposteromedial thalamic nucleus	•	•	++	++	++
Posterior thalamic nucleus	•	•	+++	+++	+++
Centrolateral nucleus	•	•	+++	+++	+++
Parafascicular nucleus	+++	+++	+++	+++	+++
Lateral geniculate nucleus, dorsal	•	•	+++	+++	+++
Lateral geniculate nucleus, ventral	•	•	++	++	++
Medial geniculate nucleus	•	•	+++	+++	+++
Lateroposterior nucleus	−	−	+++	+++	+++
**Habenula**	++	++	+++	+++	+++
**Hypothalamus**					
Preoptic nuclei	+++	+++	+++	++	++
Magnocellular preoptic nucleus	•	•	+	++	++
Anterior hypothalamic area	+++	++	++	++	+++
Lateral hypothalamic area	•	•	++	++	++
Arcuate nucleus	++	++	++	++	++
Ventromedial hypothalamic nucleus	+++	+++	+++	++	+++
Dorsomedial hypothalamic nucleus	+++	+++	+++	++	++
Tuberomammillary nucleus	•	•	++	+++	++
Premammillary nucleus	++	++	++	++	++
Medial mammillary nucleus	+++	+++	+++	+++	+++
Lateral mammillary nucleus	+	+	+	+++	+++
Magnocellular nucleus of lateral hypothalamus	•	•	−	−	++
Zona incerta	++	++	++	+	+
**Brainstem**					
**Reticular formation and related tegmental nuclei**					
Medullary reticular formation	•	•	+	+	+
Gigantocellular reticular nucleus	+	+	+	+	+
Paragigantocellular reticular nucleus	•	•	−	−	−
Intermediate reticular nucleus	•	•	−	−	−
Parvocellular reticular nucleus	−	−	−	−	−
Pontine reticular formation	−	−	−	−	−
Dorsomedial tegmental field	•	•	−	−	−
Laterodorsal tegmental nuclei	•	•	+++	•	+++
Dorsal tegmental nucleus	+++	+++	+++	•	+++
**Monoaminergic systems**					
Dorsal raphe nucleus	++	++	++	+++	+++
Locus coeruleus	+++	+++	+++	+++	+++
Ventral tegmental area	+++	+++	+++	+++	+++
**Nuclei associated with respiratory, cardiovascular and other autonomic functions**					
Nucleus of the solitary tract	++	+++	+++	+++	+++
Parabrachial nucleus	−	−	−	−	−
Dorsal motor nucleus of vagus	−	++	+++	+++	+++
Ambiguus nucleus	•	•	+++	+++	+++
**Orofacial motor nuclei**					
Motor trigeminal nucleus	+++	++	+++	+++	++
Facial nucleus	+++	++	+++	+++	+++
Hypoglossal nucleus	++	++	++	•	++
**Nuclei belonging to the somatosensory system**					
Gracile/cuneate nucleus	++	+++	+++	++	++
Principal sensory trigeminal nucleus	−	−	−	−	−
Spinal trigeminal nucleus	+++	+++	++	++	+
**Nuclei belonging to the auditory system**					
Cochlear nuclei	+++	++	+++	++	++
Lateral superior olive	−	−	−	−	−
Nucleus trapezoid body	−	−	−	−	−
Nuclei of the lateral lemniscus	−	−	−	−	−
Inferior colliculus	++	++	+	−	−
**Nuclei belonging to the visual system**					
Superior colliculus	+++	+++	++	++	+
Parabigeminal nucleus	−	−	−	−	−
**Vestibular and precerebellar nuclei**					
Vestibular nuclei	+++	+++	++	+++	++
Pontine nuclei	+	+	+	+	−
Inferior olivary nucleus	+++	+++	+++	+++	+++
Red nucleus	−	−	−	−	−
**Cerebellum**					
Deep nuclei	+	++	++	++	+
Purkinje cells	−	−	−	−−	−
Golgi cells	−	+	++	+++	+++
Granule cells	+	+	+	++	−

1 +++, strong; ++, moderate; +, weak; −, signals very low or below the threshold limit of detection; np, not present; •, not clearly identifiable.

SK1 and SK3 transcripts are detectable in the cortical neuroepithelium at E15, with both transcripts expressed at moderate to strong levels in neurons of the primordial plexiform layer (Fig. 8A,B,I,J). At E17, SK1 displays moderate expression in the subventricular zone and a weak expression in the cortical plate, suggesting an early expression in layer V–VI neurons (Fig. 8C,D, Table1), which display moderate levels of SK1 transcripts during postnatal development (P12 and P24; Fig. 10J,M, Table4) and in the adult neocortex. The SK1 overall expression pattern in the neocortex partially overlaps the SK2 pattern. In contrast, the expression pattern of SK3 in the neocortex at E17 is substantially different from that of the other two SK subunits, displaying moderate to strong signals in the subventricular zone, intermediate zone, and cortical plate (Fig. 8K,L, Table3). At E19 and E21, SK3 expression is preserved in the subventricular and intermediate zones (Fig. 9C,F, Table3), and it is still evident in the cortical plate and subplate at moderate levels at P1 and at strong levels at P3 (Fig. 10C,F, Table6). At P1 and P3, SK3 expression is strong also in layers V and VI (Fig. 10C,F, Table6). At P6 (Fig. 10I) and P2 (Fig. 10L), moderate levels of SK3 transcripts are confined to layers II–III, but they decrease by P24 (Fig. 10O, Table6), resembling the weak expression of SK3 detected in the adult neocortex (Stocker and Pedarzani, 2000).

In the piriform cortex, expression of SK1 and SK2 transcripts is detectable at E19 as moderate to strong signals (Fig. 3A,B, Tables1, 2) and persists without major changes throughout most postnatal development (Figs. 5, 6, Tables4, 5), with a decline at P24 toward the weak to moderate levels observed in adulthood (Stocker and Pedarzani, 2000). SK3 transcripts are present at E19 (Fig. 3C, Table3) and increase progressively, reaching a peak of expression between P1 and P6 (Table6). They subsequently decline to a moderate level, maintained from P12 to adulthood (Table6; Stocker and Pedarzani, 2000). Changes in expression such as those reported here and below, albeit small in some cases, were consistently observed at the cellular level of resolution in more sections. Their actual functional significance remains to be established.

At E15, the subicular neuroepithelium displays moderate levels of SK2 (Table2) and weak expression of SK1 and SK3 transcripts (Tables1, 3). At E17, as the cortical plate invades the hippocampal primordium (Bayer and Altman, 1995), all three SK channel subunits are strongly expressed in the subicular differentiating field (Fig. 8C,G,K, Tables3), with strong signals for SK2 in differentiating cells likely to become deep neurons of the para-, pre-, and subicula proper (Fig. 8G). The expression of all three SK transcripts persists at moderate (SK1, SK3) to strong levels (SK2) at E19 and E21 (Fig. 3, Tables3). After birth, the subiculum displays strong signals for SK1 (Fig. 5, Table4) and SK2 (Fig. 6, Table5) transcripts, with SK3 levels increasing from moderate to strong (Fig. 7, Table6), and this expression pattern is preserved in all parts of the adult subiculum (Stocker and Pedarzani, 2000).

The neuroepithelium of the hippocampal primordium was visible from E15 as a bulge in the medial telencephalic wall, displaying a moderate expression of SK2 (Fig. 8E, Table2) and a weak expression of SK1 (Fig. 8A, Table1) and SK3 transcripts (Fig. 8I, Table3). At E17, the cortical plate invades the hippocampal primordium, and strong expression of SK2 can be observed in differentiating neurons, most likely belonging to the CA3 layer at this stage (Fig. 8G, Table2). Strong expression of SK1 (Fig. 8C, Table1) and SK3 (Fig. 8K, Table3) transcripts is also present in differentiating hippocampal neurons at this stage. At E19 and E21, expression of all three SK subunits in the forming CA layers is clear, with strong (SK2, Fig. 1B,E; SK1, Fig. 1A,D) and moderate (SK3, Fig. 1C,F) signals (Tables3). In the postnatal period, P1–P24, strong SK1 and SK2 signals develop in the CA1–CA3 layers, (Tables4, 5) but SK3 displays an overall weak expression, which seems to have a transient strong peak at about P12 (Table6). In the dentate gyrus granule cells, which are largely generated after birth, expression of SK1 and SK2 transcripts is weak (Tables4, 5), but SK3 mRNA increases and reaches high levels starting from P6 (Table6).

#### Basal ganglia

The basal telencephalic structures display a striking differential expression of the three SK channel transcripts during embryonic development. SK1 transcripts are detectable as strong signals (Figs. 3A,D, 8A,C, Table1) and SK2 transcripts as moderate (Figs. 3B,E, 8E,G, Table2) between E15 and E19 in the striatum and pallidum, with SK2 levels declining between E15 and E19. SK3 is below the detection limit or weak throughout embryonic development in these regions (Figs. 3C,F, 8I,K, Table3). However, during postnatal development, the expression pattern of SK channel transcripts in the basal ganglia radically changes. SK1 mRNA persists at high levels only in the entopeduncular nucleus, persists at moderate levels in the caudate-putamen (Fig. 5), and drops from moderate to weak expression between P6 and P12 in the nucleus accumbens (Table4). SK2 transcripts are present at moderate levels only in the entopeduncular nucleus, and they are weakly expressed in the caudate-putamen and absent or weak in all other basal ganglia structures after birth (Fig. 6, Table5). By contrast, the expression of SK3 transcripts is clearly upregulated during postnatal development, being strong in the caudate-putamen (e.g., Fig. 7F) and in the nucleus accumbens (e.g., Fig. 7I) and moderate in the globus pallidus (Table6).

#### Thalamus

Expression of all three SK channel subunits is detectable in the anterior and posterior thalamic differentiating fields starting from E17, with SK2 and SK3 transcripts expressed strongly and SK1 at moderate levels (Fig. 2M–O, Tables3). At E19 and E21, strong expression of SK1 is present in the anterior thalamus, with moderate to weak signals in the intermediate and posterior thalamus (Fig. 3A,D, Table1). Moderately strong expression of SK2 transcripts is detectable in all thalamic differentiating areas at these developmental stages (Fig. 3B,E, Table2), whereas SK3 transcripts show a decrease, from strong to moderate levels, between E19 and E21 in the anterior and intermediate thalamic areas (Fig. 3C,F, Table3). During postnatal development, SK1 shows strong expression, which is maintained from P1 to P24 in the reticular thalamic nucleus and the anterodorsal, anteroventral (Fig. 5H), mediodorsal, and paraventricular nuclei (Table4). SK1 transcripts show instead a progressive decrease during postnatal development in the ventrolateral, central medial, ventromedial, ventroposterolateral, ventroposteromedial (Fig. 5D–F), posterior, lateroposterior, and centrolateral nuclei (Table4). Similarly, SK1 levels decline from P6 to P24 in the dorsolateral and medial geniculate nuclei (Table4). SK2 shows its strongest expression in the reticular thalamic nucleus, where it does not change throughout postnatal development (Figs. 6H,I, 12, Table5). Strong signals for SK2 transcripts are also present in the anterodorsal, ventroposterolateral, and ventroposteromedial nuclei (Table5). SK3 signals are strong in several thalamic nuclei throughout postnatal development. These include the anterodorsal (Fig. 7G), anteromedial, laterodorsal, anterior paraventricular, lateroposterior, posterior, centrolateral, and parafascicular nuclei in addition to the dorsolateral and medial geniculate nuclei (Fig. 7, Table6). In most thalamic nuclei (Fig. 7B,H,I) signals for SK3 expression appear constant during postnatal development, with the exception of the anteroventral, central medial, and lateroposterior nuclei, where an increase in expression is observed between P1 and P24 (Table6).

#### Cerebellum and precerebellar nuclei

Cerebellar neurons are generated and migrate at different time points, with the deep cerebellar nuclei neurons appearing first, with a neurogenesis peak at about E14; the Purkinje cells of the cerebellar cortex immediately following, with a peak at about E15; and Golgi cells appearing between E19 and P2, whereas granule cells have a late time of origin at P8–15 (Bayer and Altman, 1995). Expression of SK channel subunits was first detected at E15 (Figs. 2, 3A–C), with moderate levels of SK1 and strong signals for SK2 transcripts present in the cerebellar differentiating field, whereas SK3 was hardly detectable at this age (Tables3). Similar expression levels for the three SK channel subunits persist at E17 (Fig. 3D–F, Tables3). At E19 and E21, SK1 expression is strong in the deep nuclei (Fig. 3G,J, Table1), and SK2 predominates in the differentiating Purkinje cells (Figs. 3B,E, 13H,K, Table2). At these stages, SK3 expression in the cerebellar region is still weak (Fig. 3I,L, Table3), and none of the SK subunits is expressed at detectable levels in the external germinal layer (Fig. 3, Tables3).

Postnatally, SK1 expression persists as strong in large neurons of the deep cerebellar nuclei (Fig. 4A) and is detectable as weak in the internal granule cell layer (Fig. 5, Table4). SK2 transcripts are strongly expressed in Purkinje cells up to P12 and subsequently decrease to moderate levels at P24 (Table5; Cingolani et al., 2002). SK2 signals increase from moderate to strong between P6 and P12 in neurons of the deep cerebellar nuclei and from weak to moderate in the internal granule cell layer (Fig. 6, Table5). Finally, SK3 is expressed at moderate levels in the deep cerebellar nuclei (Fig. 4B, Table6). In the cerebellar cortex, SK3 signals progressively increase from weak to strong in the Golgi cells (Fig. 4C–F, Table6), but they are weak in the granule cells (Fig. 4C–F, Table6).

At the level of the precerebellar nuclei, moderate levels of SK1 transcript (Fig. 3A) and a strong expression of SK2 (Fig. 3B) can be observed in the differentiating anterior and posterior pons at E15 (Tables1, 2). At this stage, SK3 transcripts are more selectively strongly expressed in the neuroepithelium and a small portion of the posterior pons (Fig. 3C, Table3). While the expression of SK3 persists as strong in the posterior pons and neuroepithelium also at E17 (Fig. 3F, Table3), the expression of SK2 in the differentiating pons drops to moderate levels at this stage (Fig. 3E, Table2), and a moderate expression is observed also for SK1 (Fig. 3D, Table1). Postnatally, the pontine nuclei display gradually increasing expression levels of SK1 transcripts (Fig. 5A,C,E,G, Table4), strong signals for SK2 throughout postnatal development (Fig. 6A,C,E,G, Table5), and weak or undetectable ones for SK3 (Table6). Despite originating from a common neuroepithelium (Bayer and Altman, 1995), neurons of the inferior olive display a different pattern of expression of SK channel subunits, with strong expression of SK1 from P1 to P6, which declines substantially at P12 (Fig. 5A,D, Table4), and SK2 (Fig. 5B,D, Table5) and SK3 (Figs. 7G, 5C,F, Table6) transcripts giving persisting strong signals throughout postnatal development.

## DISCUSSION

This study shows that SK channels are expressed at early stages of development of the CNS. The three SK channel subunits display different developmental expression gradients in distinct CNS regions, with time points of expression and up-or downregulation that can be associated with a range of diverse developmental events, such as for example neurogenesis and differentiation. In the case of SK1 and SK2, strongest expression at embryonic stages of development was observed in the undifferentiated cortex and hippocampus starting from E15 and was maintained throughout postnatal development and in the adult brain (Stocker and Pedarzani, 2000; Sailer et al., 2004). By contrast, the SK3 subunit shows strikingly strong expression in the undifferentiated cortex and hippocampal formation during embryonic development, which decreases in the adult (Stocker and Pedarzani, 2000). In particular, strong signals for SK3 were observed in the subventricular zone up to E19–E21 and in the intermediate zone up to E17, suggesting a link to regulation of the proliferation of neuronal precursor cells and early neuronal differentiation (Bayer and Altman, 1995). Further supporting this hypothesis, SK3 is expressed predominantly in the neuroepithelia (e.g., cochlear and precerebellar). This is in agreement with recent studies proposing a role for SK3 channels in early neurogenesis and differentiation of neuronal progenitor cells (Liebau et al., 2011).

SK1 and SK2 transcripts also showed strong expression starting from E15 in the striatum, where SK3 was instead not detectable until after birth. While SK1 and SK2 decreased throughout pre-and postnatal development, SK3 strongly increased during postnatal development, being the predominant SK channel subunit expressed in the caudate-putamen and nucleus accumbens after birth and in the juvenile and adult rat brain (Stocker and Pedarzani, 2000). Neurons of the anterior thalamic complex are generated mainly on E16 and E17 and settle about 3 days later (Bayer and Altman, 1995), when SK1 and SK3 subunits display strong expression, maintained throughout postnatal development and in the adult brain (Stocker and Pedarzani, 2000). Finally, trigeminal ganglion neurons are born between E9.5 and E14.5 and innervate their target regions and establish receptive fields that, by E16, are similar to those in adult animals (Waite, 2004). All three SK channel transcripts are strongly expressed in the trigeminal ganglion as early as E15, with SK1 and SK2 persisting at strong levels until birth. Conversely, SK3 expression declines at E17 and E19 following the establishment of receptive fields.

During postnatal development a striking overall pattern emerges for the three SK channel transcripts, whereby the SK2 subunit is expressed at comparable levels as in the adult brain in most regions starting from P1. Few exceptions are represented by the facial nucleus, where SK2 expression increases from P1 to P12, and the cochlear nucleus and Purkinje neurons, where SK2 expression decreases from P1 to P24 (Cingolani et al., 2002). By contrast, both SK1 and SK3 show major changes in expression during the perinatal and postnatal period compared with the adult age in several brain regions (Tables4, 6; Stocker and Pedarzani, 2000). As observed in the adult brain, expression patterns of SK1 and SK2 subunits overlap throughout postnatal development and coevolve in several brain regions, whereas SK3 displays a rather distinct and complementary expression pattern.

The Allen Brain Atlas (Lein et al., 2007; http://www.brain-map.org) and the GENSAT database (Gong et al., 2003; http://www.gensat.org) provide extensive online collections of gene expression maps for nearly all genes expressed in the adult and developing mouse brain. The GENSAT database does not report any information on SK channels, but the expression of all three SK channel subunits has been analyzed by nonradioactive in situ hybridizations in the Allen atlas. SK1 and SK2 are shown to have low levels of expression limited to a few regions of the adult mouse CNS, with SK3 displaying instead ubiquitous expression. The expression levels reported for SK1 and SK2 in the adult rat brain are stronger and more widespread (Stocker and Pedarzani, 2000). An even stronger discrepancy is evident for SK3, which displays a predominantly and rather restricted subcortical expression pattern, as shown in in situ hybridization studies on the adult rat brain (Stocker and Pedarzani, 2000; Tacconi et al., 2001) and by immunohistochemistry in the mouse (Sailer et al., 2004) and rat (Tacconi et al., 2001) brain. This difference could be a consequence of the use of riboprobes when dealing with highly homologous transcripts (Allen Atlas), a problem prevented by the use of shorter and more specific oligonucleotides (Wisden and Morris, 1994; Stocker and Pedarzani, 2000; Tacconi et al., 2001). For the developing mouse brain, the Allen Atlas provides information only on the distribution on the SK2 channel transcript, with little to no expression detectable during embryonic development. The early expression of all SK subunits in rat embryonic development reported in this study suggests an involvement in the regulation of developmental processes. Apamin is a bee venom toxin that binds and selectively inhibits SK channels (for review see Pedarzani and Stocker, 2008). Our findings are consistent with the results of apamin-binding studies, showing labeling of E16–E17 neuronal cultures (Seagar et al., 1984) and proliferative periventricular zones before birth and of cortex and hippocampus already at birth (Mourre et al., 1987).

The expression maps of the three SK channel transcripts during pre-and postnatal development raise questions concerning the potential function and subunit composition of SK channels at various developmental stages. There are only a few studies investigating the function of pharmacologically defined apamin-sensitive Ca^2+^-activated K^+^ currents in developing neurons, most of which support a role of SK channels in the early regulation of membrane excitability and firing properties. This is the case for neurons in the medial vestibular nucleus, where SK channels counteract Na^+^ and Ca^2+^ currents underlying the generation of depolarizing plateau potentials shortly after birth (P5; Dutia and Johnston, 1998) and where all three SK channel subunits are expressed as early as at birth (this study). Similarly, in neocortical neurons of layers II/III and V, an apamin-sensitive current contributes to the medium-duration afterhyperpolarization at P7 and persists with similar properties until adulthood (Lorenzon and Foehring, 1993). This is in good agreement with the expression of SK channel subunits detected from P6 in layer II/III and as early as P1 in layer V neurons (this study). In phrenic motoneurons, apamin-sensitive Ca^2+^-activated K^+^ currents were measured as early as at E18, when they contributed to shaping firing and afterpotentials upon the start of inspiratory drive transmission (Martin-Caraballo and Greer, 2000). Also in this case, the functional expression of SK channels in motoneurons matches the early expression of SK1–3 mRNAs detected in the spinal cord (this study). Finally, the presence of the SK2 channel transcript in the mouse hippocampal formation has been shown by real-time PCR starting from P0 and progressively increasing during the first 3 weeks of life, in agreement with our findings in the rat hippocampus. Protein expression follows a similar trend, with SK2-containing channels at synaptic locations increasing from P5 to P30 (Ballesteros-Merino et al., 2012).

The largely overlapping expression patterns of SK channel subunits, particularly SK1 and SK2, starting from prenatal developmental stages, suggest the formation of heteromeric channels, formed by two or even three different SK channel subunits. Heteromeric channels are formed upon coexpression of recombinant SK channel subunits in heterologous expression systems (Ishii et al., 1997; Benton et al., 2003; Monaghan et al., 2004), but their existence in native systems is still uncertain with biochemical evidence arguing in favor and against (Sailer et al., 2002; Strassmaier et al., 2005; Tuteja et al., 2010) and immunohistochemical studies suggesting subcellular segregation of different SK subunits (Sailer et al., 2002; Sailer et al., 2004). In particular, the role of the SK1 subunit is still unclear. Unlike the human homologous SK1 subunit, the rat SK1 (rSK1) does not form functional homomeric SK1 channels in heterologous expression systems (Benton et al., 2003; D'hoedt et al., 2004). Chimeric rSK1 channels are capable of membrane expression and are insensitive to the specific SK channel toxin inhibitor apamin (D'hoedt et al., 2004), unlike their human counterparts. Putative rSK1/rSK2 heteromeric channels in heterologous systems display a sensitivity to apamin (Benton et al., 2003; Weatherall et al., 2011) comparable to that observed for the native, SK-mediated current in CA1 pyramidal neurons of young adult rats (Stocker et al., 1999), suggesting that heteromeric rSK1/rSK2 channels are formed in these neurons.

In heterologous expression systems, the coexpression of rSK1 and rSK2 subunits leads to larger currents (Benton et al., 2003), whereas coexpression of rSK1 and rSK3 subunits results in smaller currents (Monaghan et al., 2004). Our findings on the ontogeny of SK channel expression, in particular the relatively stable levels of SK2 associated in several brain regions with decreasing or increasing levels of SK1 during perinatal and postnatal development, might result in significant changes in the functional expression of SK-mediated currents. Similar regulatory mechanisms might take place in neurons coexpressing SK1 and SK3 and displaying relative changes in the ratio of these two transcripts. Whether this finely tuned regulation of SK channel transcript coexpression in developing neurons has a functional consequence requires future work using electrophysiology in combination with pharmacological tools specific for channels with defined subunit compositions, to shed light on the molecular makeup and function of SK channels in neurons at different developmental stages.

A further potential source of developmentally regulated diversity in the molecular makeup of SK channels is suggested by the distinct developmental time courses of embryonic and postnatal expression of multiple transcripts observed in the Northern blot analysis. The presence of a single gene for each SK subunit in the rat genome (for review see Stocker, 2004) suggests that the multiple bands observed in the Northern blot are the consequence of alternative splicing and the use of alternative promoters. Indeed, both alternative splicing (Shmukler et al., 2001; Kolski-Andreaco et al., 2004; Wittekindt et al., 2004) and the use of alternative promoters (Strassmaier et al., 2005) have been described for SK subunits. A detailed study on specific SK channel splice variants and their functional correlates in developing neurons is still needed.

SK channels are the center of multiprotein signaling complexes that, in addition to the pore-forming SK channel subunits, include calmodulin as a constitutively bound calcium sensor and casein kinase and protein-phosphatase 2A as closely associated enzymes modulating the calcium sensitivity of the channels (Bildl et al., 2004; Allen et al., 2007). Calmodulin transcripts have been detected in the rat brain as early as E14 by Northern blot analysis (Weinman et al., 1991), with mRNAs from different calmodulin-encoding genes showing region-specific developmental expression patterns (Ni et al., 1992). Calmodulin immunoreactivity has been found in the neural tube of developing mouse embryos as early as E9.5 (Seto-Ohshima et al., 1987), and in the rat brain it was detected at P1 and shown to increase during the first 2 postnatal weeks (Berry and Brown, 1995). Thus, based on these ontogenetic studies, expression of calmodulin most likely is not a temporal limiting step for the assembly of functional SK channels at embryonic and postnatal neuronal developmental stages. Similarly to calmodulin, the casein kinase 2 alpha subunit (CK2α) is expressed at very early stages of embryonic neuronal development (Dominguez et al., 2011). A second gene encoding for the casein kinase 2 alpha subunit (CK2α') appears at a later developmental stage, at the time of dendrite maturation and synaptogenesis (Diaz-Nido et al., 1994; Moreno et al., 1999). Finally, the regulatory subunit casein kinase 2 beta (CK2β) is essential at very early stages of embryonic development for proliferation and differentiation of neural progenitor cells (Huillard et al., 2010). Protein phosphatase 2A (PP2A) is already expressed during gastrulation and neurulation (Gotz and Kues, 1999). PP2A regulatory subunits instead display different expression patterns, with Bα and Bβ detectable in embryonic brains, whereas Bγ increases sharply after birth (Strack et al., 1998). Based on these studies, it is likely that the SK channel multiprotein signaling complexes observed in the adult brain form already during embryonic development, when the SK channel subunits first appear.

In the adult brain, SK channels are activated by intracellular calcium elevations whose origin varies from cell type to cell type, or even within different subcellular compartments in the same neuron (for review see Pedarzani and Stocker, 2008). The calcium sources that are functionally coupled to the activation of SK channels in neurons include voltage-gated Ca^2+^ channels, N-methyl-D-aspartate (NMDA) glutamate receptors, and ryanodine-sensitive and IP_3_-sensitive stores (for review see Adelman et al., 2012). Most neuronal voltage-gated Ca^2+^ channels (Ca_V_1.2, Ca_V_1.3, Ca_V_2.1, Ca_V_2.2, Ca_V_2.3, Ca_V_3.1, and Ca_V_3.3) are expressed in different brain regions during early embryonic development (Vance et al., 1998; Meacham et al., 2003; Yunker et al., 2003; Schlick et al., 2010). Similarly, several NMDA receptor subunits are expressed during embryonic development of the CNS (Monyer et al., 1994). Channels responsible for the release of calcium from intracellular stores, such as IP_3_ receptors (Dent et al., 1996; Faure et al., 2001) and ryanodine-sensitive channels (Faure et al., 2001), have been found in neurons as early as at stage E11. For all these calcium-permeable channels, changes in expression levels, subunit composition, or subcellular distribution have been reported in the course of the perinatal and postnatal development of the nervous system. At which stage SK channels are first functionally coupled to these calcium sources and how this coupling evolves in the course of neuronal development are still largely unresolved issues.

In conclusion, this study reports an early expression of SK channel subunit transcripts, starting from E12. If channel translation and translocation to the membrane follow shortly after the appearance of transcripts, SK channels are ideally suited to respond to the calcium transients that have been implicated in various stages of neuronal development, including proliferation, migration, differentiation, and survival (Rosenberg and Spitzer, 2011). In *Drosophila*, different types of voltage-and calcium-dependent potassium channels have been shown to regulate the size and dynamics of calcium transients in different subcellular compartments, influencing activity-dependent neuronal differentiation (Berke et al., 2006). We propose that, given their early and wide expression, sensitivity to calcium, and roles played in mature neurons, SK channels might play a key role in shaping the temporal dynamics and spatial spread of early calcium transients in mammalian neurons, thereby influencing the specific effects of calcium signaling in neuronal development.

## ROLE OF AUTHORS

All authors had full access to all the data in the study and take responsibility for the integrity of the data and the accuracy of the data analysis. Study concept and design: PP, MS. Study supervision: PP, MS. Acquisition of data: MG, LAC. Analysis and interpretation of data: MG, LAC, PP, MS. Drafting of the manuscript: PP, MS. Obtained funding: PP, MS.

## Abbreviations

I–III: alternative layer II/III
IV: alternative layer IV
V: alternative layer V
VI: alternative layer VI
4V: 4th ventricle
5Gn: trigeminal ganglion
Acb: accumbens nucleus
AD: anterodorsal thalamic nucleus
AM: anteromedial thalamic nucleus
Amg: amygdala
AO: anterior olfactory nucleus
AV: anteroventral thalamic nucleus
BST: bed nucleus of the stria terminalis
CA1: field CA1 of Ammon's horn
CA2: field CA2 of Ammon's horn
CA3: field CA3 of Ammon's horn
Cb: cerebellum
cb: cerebellar neuroepithelium
CGPn: central gray of pons
CN: cochlear nucleus
CP: cortical plate
CPu: caudate putamen (striatum)
Cx: cerebral cortex
cx: cortical neuroepithelium
dfu: dorsal funiculus of spinal cord
DH: dorsal horns of spinal cord
Dien: diencephalon
DN: deep cerebellar nuclei
DRG: dorsal root ganglion/a
EGL: external germinal layer of cerebellum
GrCb: granule cell layer of the cerebellum
Hb: habenula
Hi: hippocampal formation
hi: hippocampal neuroepithelium
hy: hypothalamic neuroepithelium
I9Gn: inferior glossopharyngeal ganglion
IC: inferior colliculus
ic: inferior colliculus neuroepithelium
Inte: intestine
IntZ: intermediate zone of spinal gray
IO: inferior olive
IZ: intermediate zone of the cerebral cortex
LS: lateral septal nucleus
Mes: mesencephalon
ML: molecular layer of the cerebral cortex
MZ: marginal zone
ne: neuroepithelium
OB: olfactory bulb
Pir: piriform cortex
Pk: cerebellar Purkinje cell layer
Pn: pontine nuclei
Pons: pons
PPL: primordial plexiform layer
Pro: prosencephalon
Rho: rhombencephalon
Rt: reticular thalamic nucleus
S: subiculum
SC: superior colliculus
sc: superior colliculus neuroepithelium
SK: small-conductance Ca^2+^-activated K^+^ channel
SNC: substrantia nigra, pars compacta
Sol: nucleus of the solitary tract
SpC: spinal cord
spn: spinal nerve
Str: striatum (neuroepithelium/diff. field)
SV: neocortical subventricular zone
SP: subplate
Telen: telencephalon
Th: thalamus
Tu: olfactory tubercle
vc: ventral commissure of spinal cord
VH: ventral horns of spinal cord
VPL: ventral posterolateral thalamic n.
VPM: ventral posteromedial thalamic n.
VTA: ventral tegmental area
ZI: zona incerta
